# Clinical and microbiological epidemiology of *Candida* infections in a high-complexity hospital in Tolima, Colombia (2014–2024)

**DOI:** 10.1371/journal.pone.0354684

**Published:** 2026-07-24

**Authors:** Rafael A. Ramírez-Trujillo, Paula Carvajal-Hernández, Ángel González

**Affiliations:** 1 Basic and Applied Microbiology Research Group (MICROBA), School of Microbiology, Universidad de Antioquia, Medellin, Colombia; 2 Sub-directorate of Health Services, Clinica Tolima, Ibagué, Colombia; Ramathibodi Hospital, Mahidol University, THAILAND

## Abstract

**Background:**

*Candida* spp. infections are an increasing challenge in high-complexity hospitals, yet epidemiological data remain scarce in underrepresented in Colombian regions such as Tolima.

**Methods:**

We conducted a retrospective observational study in a high-complexity hospital in Ibagué (Tolima, Colombia) from 2014 to 2024, integrating two institutional data sources: administrative/clinical records and the microbiology laboratory database (WHONET). Species identification relied on culture and VITEK, and antifungal susceptibility was interpreted using criteria from the Clinical and Laboratory Standards Institute (CLSI) and the European Committee on Antimicrobial Susceptibility Testing (EUCAST). We summarized data using frequencies/proportions and medians (IQR), explored patterns with multiple correspondence analysis (MCA), and estimated associations with candidemia using penalized multivariable logistic regression due to low event frequency.

**Results:**

We identified 987 candidiasis episodes and 776 fungal isolates, of which 314 were *Candida* (40.46%). Mucocutaneous disease predominated (vulvovaginal 50.7%; oropharyngeal 24.3%), while candidemia represented 2.0% of episodes. Among isolates, *Candida albicans* was most frequent (58.9%), followed by *C. parapsilosis* (16.6%), *C. tropicalis* (12.1%), and *Nakaseomyces glabratus* (6.4%); *Candida auris* was detected once. In exploratory clinical/administrative models, clinically recorded candidemia showed associations with invasive devices (OR 5.54, 95% CI 2.01–15.64), recent surgery (OR 7.11, 95% CI 1.20–30.88) and tumor (OR 19.88, 95% CI 3.14–97.51). Susceptibility data were available for 196/314 isolates (62.4%); echinocandin activity was high, whereas azole susceptibility was more variable.

**Conclusions:**

Candidiasis showed a sustained recorded burden and substantial non-*albicans* diversity, supporting local surveillance, species-level identification, and isolate-level susceptibility testing.

## Introduction

Fungal infections (FIs) are an increasing public health problem, causing 1.5 million deaths annually, a burden comparable to tuberculosis or malaria [1]. This rise reflects expanding vulnerable populations (e.g., diabetes, untreated HIV, cancer, transplantation, immunosuppressive therapies) and healthcare exposures in critically ill patients, including broad-spectrum antibiotics and invasive devices [2]. *Candida* spp., commensal yeasts, can act opportunistically, producing disease from superficial mucocutaneous infection to invasive candidiasis and candidemia, a frequent cause of hospital-acquired sepsis, particularly in intensive care units (ICUs) [3,4]. Globally, a shift toward non-*albicans* species and variable antifungal susceptibility complicates empiric therapy and may worsen outcomes [5].

In 2022, the World Health Organization (WHO) issued its first list of priority fungal pathogens, highlighting *C. auris* and *C. albicans* as critical and *Nakaseomyces glabratus*, *C. tropicalis*, and *C. parapsilosis* as high-priority pathogens. Despite surveillance initiatives in several regions, data in Latin America and especially Colombia, remain limited and fragmented, with underreporting exacerbated by the absence of mandatory notification for most fungal diseases [2,6]. Recent actions led by the Colombian National Institute of Health (INS), including the incorporation of *C. auris* into Colombia’s National Public Health Surveillance System (SIVIGILA, for its Spanish acronym), the updating of national guidelines, and laboratory surveillance criteria for *Candida* spp. isolates recovered from healthcare-associated infections (HAIs; IAAS in Spanish) or showing antifungal resistance profiles, represent important progress in fungal disease surveillance; however, implementation and data availability remain incomplete [7].

In Tolima, evidence on local prevalence, species distribution, and resistance patterns of *Candida* spp. is scarce relative to other Colombian regions [2,8], limiting data-driven decisions and detection of temporal changes or intrahospital events [9,10]. Therefore, this single-center study aimed to evaluate the epidemiology, species distribution, exploratory associated factors, and antifungal susceptibility profiles of *Candida* infections at a high-complexity referral hospital in Ibagué, Tolima, Colombia, spanning a ten-year period (2014–2024).

## Methodology

### Design and setting

An observational, retrospective descriptive-analytical study was conducted in a high-complexity hospital in Ibagué, Tolima, Colombia, between 2014 and 2024. Two institutional data sources from the same participating hospital were used: (A) a clinical/administrative database derived from hospital administrative and clinical records, and (B) a microbiological database derived from Microbiology Laboratory records. Although both sources came from the same physical institution, they were generated through separate institutional information workflows and were not linked record by record for this retrospective study. The primary scope of the study was the overall clinical and microbiological burden of *Candida* infections, including mucocutaneous, localized, invasive, and bloodstream presentations, rather than candidemia alone.

MIC-based susceptibility analyses were restricted to 2022–2024, the period with structured MIC exports in WHONET format. The clinical/administrative database was used to describe clinically recorded candidiasis episodes and to explore factors associated with candidemia and invasive candidiasis, whereas the microbiological database was used to describe *Candida* spp. isolates, specimen sources, species distribution, antifungal susceptibility, and factors associated with non-*albicans* isolation. Selection-stage counts and data access details are provided in (S1 Text). Data were accessed for research purposes on 2 August 2025 for the clinical/administrative database and on 9 August 2025 for the microbiological database. This study is reported in accordance with the STROBE statement and the RECORD extension for routinely collected health data; completed checklists are available as Supporting Information (S1 Checklist).

### Population, definitions, and selection

Patients with a clinical diagnosis of candidiasis and/or microbiological identification of *Candida* spp. were included. The unit of analysis was an infection episode/record in the hospital database and an isolate record in the laboratory database. Exact duplicates were removed within each database; no additional time-window deduplication was applied. Records without clinical or microbiological corroboration, contaminated samples, or missing key variables were excluded. Clinical forms were classified using the recorded clinical/administrative diagnosis and ICD-10 coding (S1 Table). Cases were grouped as mucocutaneous/localized non-invasive candidiasis, candidemia, deep-seated non-candidemic invasive candidiasis, or other/unspecified candidiasis. Deep-seated non-candidemic invasive candidiasis included organ-specific invasive records without recorded candidemia, including CNS involvement/meningitis. Combined or disseminated candidiasis was not assigned as a separate category because it was not documented in the clinical/administrative diagnosis fields. For suspected invasive fungal infection, EORTC/MSG categories (proven/probable/possible) were applied when appropriate.

### Isolation, identification, and susceptibility

Clinical samples were processed by the institution’s Microbiology Laboratory as part of routine care. *Candida* spp. were identified using routine laboratory methods, including conventional culture and/or BD Phoenix during 2014–2021 and the VITEK 2 Compact system during 2022–2024. During the VITEK 2 Compact period, yeast identification was performed using the YST card, and antifungal susceptibility testing was performed using the AST-YS08 card. WHONET MIC data exports (µg/mL) included amphotericin B, fluconazole, voriconazole, caspofungin, micafungin, and flucytosine (5-FC). MICs were interpreted using current CLSI guidance (S/I/R) when breakpoints were available. Because clinical breakpoints are not available for 5-FC for several species, 5-FC was analyzed exclusively using EUCAST ECOFF classification and reported as wild-type (WT) or non-wild-type (NWT), as described in [11] (S2 Table, S2 Text). To avoid overinterpretation, 5-FC results are reported separately in S6 Table. Isolates without MIC results for a given antifungal were excluded from that antifungal-specific analysis (S3 Table).

### Statistical analysis

Data were prepared, cleaned, coded, and managed in Microsoft Excel before statistical analysis. All statistical analyses were performed using R software, version 4.4.2. Categorical variables were summarized as n (%) and continuous variables as median (IQR). Group comparisons used χ² tests and non-parametric tests; two-sided *p value* <0.05 was considered significant. Multiple correspondence analysis (MCA) was applied to selected categorical variables defined a priori. Logistic regression estimated odds ratios (ORs) and 95% confidence intervals (CIs); Firth-penalized logistic regression was used for candidemia (infrequent outcome), and standard logistic regression for invasive candidiasis and the non-albicans outcome. Age was categorized as <27 vs ≥ 27 years, and length of stay as 1–3, 4–10, and ≥11 days (reference: ≥ 11). Missing data were handled by reporting variable-specific denominators; regression models used complete-case analysis. Regression models were exploratory and data-source specific: candidemia and invasive candidiasis models used the clinical/administrative database, whereas the non-*albicans* model used microbiological isolate records. Because the databases were not linked record by record, these models were not interpreted as patient-level clinical–microbiological risk models.

### Ethics statement

The research ethics committee of the participating institution approved this study (Acta 116, 24 January 2025). This retrospective study used secondary clinical and microbiological records obtained under restricted institutional access. For public sharing, only de-identified datasets without direct personal identifiers or sensitive institutional variables were deposited in Zenodo. The study was conducted in accordance with the Declaration of Helsinki, CIOMS guidelines, and applicable Colombian regulations, including Resolution 8430 of 1993 for research without risk and provisions regarding the management of medical records. The requirement for informed consent was waived by the ethics committee because the study used retrospective, de-identified data. No animal studies were performed.

## Results

### Institutional burden and temporal trend (2014–2024)

**Fig 1:**
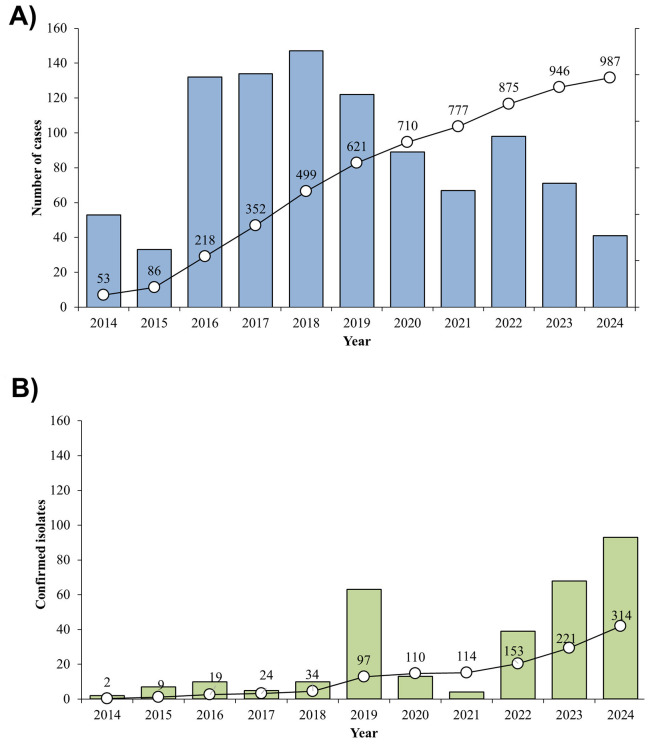
Temporal trend and cumulative counts of clinically recorded candidiasis episodes and microbiologically confirmed *Candida* isolate records, 2014–2024. **(A)** Probable candidiasis episodes recorded in the hospital clinical/administrative database. **(B)** Microbiologically confirmed *Candida* spp. isolate records from the laboratory database. In both panels, bars and their numeric labels represent annual counts, whereas the line and its numeric labels represent cumulative counts over time.

During 2014–2024, the hospital administrative database included 243,013 cumulative institutional care records across different service locations. This administrative denominator should not be interpreted as a count of unique patients. The clinical database identified 987 episodes compatible with candidiasis, corresponding to a recorded candidiasis burden of 0.41% (4.06 per 1,000 institutional care records). Annual rates ranged from 0.22% to 0.61%, peaking in 2018 (n = 147) and decreasing thereafter, while cumulative counts increased over time (Fig 1A).

In the microbiological database, 776 fungal/yeast isolate records were available, of which 314 were Candida spp. (40.46%, 314/776). Because laboratory data are recorded as isolates, counts may exceed clinical episodes due to repeat cultures. Cumulative confirmed isolates increased over time (Fig 1B).

### Clinical spectrum and population characteristics (clinical database; n=987)

**Fig 2:**
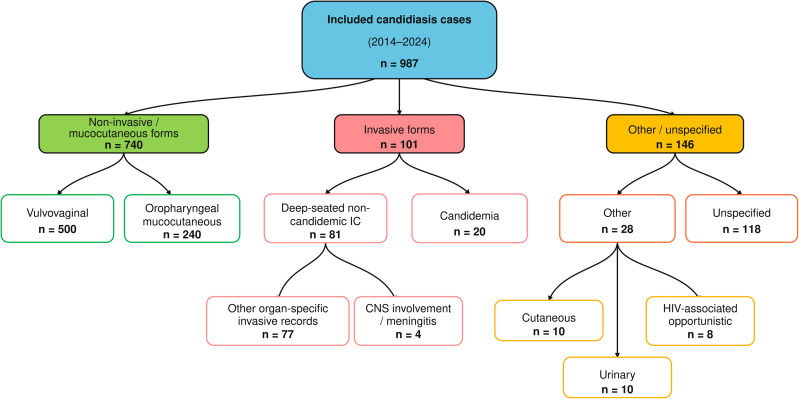
Operational classification of clinically recorded candidiasis episodes, 2014–2024. IC, invasive candidiasis; CNS, central nervous system. Deep-seated non-candidemic IC comprised organ-specific invasive records, including CNS involvement/meningitis.

The clinical spectrum was dominated by mucocutaneous disease: vulvovaginal candidiasis 50.66% (n = 500) and oropharyngeal candidiasis 24.32% (n = 240). Other categories included unspecified candidiasis 11.96% (n = 118), deep-seated non-candidemic invasive candidiasis 8.21% (n = 81), and candidemia 2.03% (n = 20) (Fig 2). The other/unspecified group (n = 146) mainly reflected unspecified candidiasis records (n = 118) and should be interpreted as evidence of limited diagnostic granularity in the retrospective clinical/administrative database rather than as a distinct clinical syndrome.

Overall, 77.81% of episodes occurred in women (F:M 3.5:1), largely driven by vulvovaginal candidiasis. Excluding this category, oropharyngeal candidiasis remained more frequent in women (61.25%) than men (38.75%). Median age was 29 years (IQR 22–53); 28 years in women (IQR 22–44) and 39 years in men (IQR 8–69). Oropharyngeal candidiasis showed a bimodal distribution, with 32.9% in early childhood (79/240) and 35.8% in those ≥60 years (86/240).

**Fig 3:**
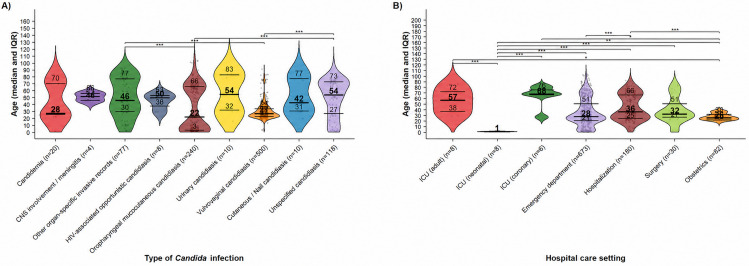
Age distribution according to A) type of *Candida* infection and B) hospital care setting, with statistical significance indicated where applicable.

Age differed by infection type (p < 0.001), with higher medians in invasive forms, including candidemia (38 years, IQR 28–70) and other organ-specific invasive records (46 years, IQR 30–77), than in vulvovaginal candidiasis (27 years, IQR 24–34) (Fig 3A). Age also differed by care setting (p < 0.001), with higher medians in critical care (coronary ICU 69 years; adult ICU 57 years) and lower medians in obstetrics (26 years) and neonatal ICU (1 year) (Fig 3B). Length of stay differed by infection type (p < 0.001), with longer stays for candidemia and deep-seated non-candidemic invasive candidiasis, including CNS involvement/meningitis, than for mucocutaneous/non-invasive forms. By service, length of stay was largely explained by care setting (η² = 0.51; *p* < 0.001), with longer stays in ICU and general wards (Table 1).

**Table 1:** Sociodemographic, clinical, and care-related characteristics of candidiasis episodes (suspected/probable) (n = 987) in a high-complexity hospital in Tolima, Colombia (2014–2024).

Variable	Categories	n	%
**Sex**	Female	768	77.81
	Male	219	22.19
**Age group**	Early childhood (0–5 years)	101	10.23
	Childhood (6–11 years)	35	3.55
	Adolescence (12–18 years)	34	3.44
	Youth (19–26 years)	239	24.21
	Adulthood (27–59 years)	372	37.69
	Older adult (≥60 years)	206	20.87
**Hospital location / care setting**	ICU (adult)	8	0.81
	ICU (neonatal)	8	0.81
	ICU (coronary)	6	0.61
	Emergency department	673	68.19
	Hospitalization	180	18.24
	Surgery	30	3.04
	Obstetrics	82	8.31
**Length of hospital stay**	Short (1–3 days)	853	86.42
	Medium (4–10 days)	85	8.61
	Prolonged (11–30 days)	37	3,75
	Extended (>31 days)	12	1,22
**Type of infection**	Candidemia	20	2.03
	CNS involvement/meningitis	4	0.41
	Other organ-specific invasive records	77	7.80
	HIV-associated opportunistic	8	0.81
	Mucocutaneous oropharyngeal	240	24.32
	Urinary	10	1.01
	Vulvovaginal candidiasis	500	50.66
	Cutaneous/nail candidiasis	10	1.01
	Unspecified candidiasis	118	11.96
**Comorbidities**	Neoplasia / tumor	11	1.11
	Diabetes mellitus	31	3.14
	Gastrointestinal disease	51	5.17
	Respiratory dysfunction	50	5.07
	Cardiovascular disease	12	1.22
	Arterial hypertension	10	1.01
	Urinary tract infection	40	4.05
	Threatened abortion	28	2.84
	Invasive devices	125	12.66

**Note:** Comorbidities are not mutually exclusive; therefore, percentages do not sum to 100%. Percentages were calculated using n = 987 as the denominator, unless otherwise specified due to missing data or not determined (ND) values.

### Species distribution, sample origin, and patterns by service (microbiological database; n = 314)

**Fig 4:**
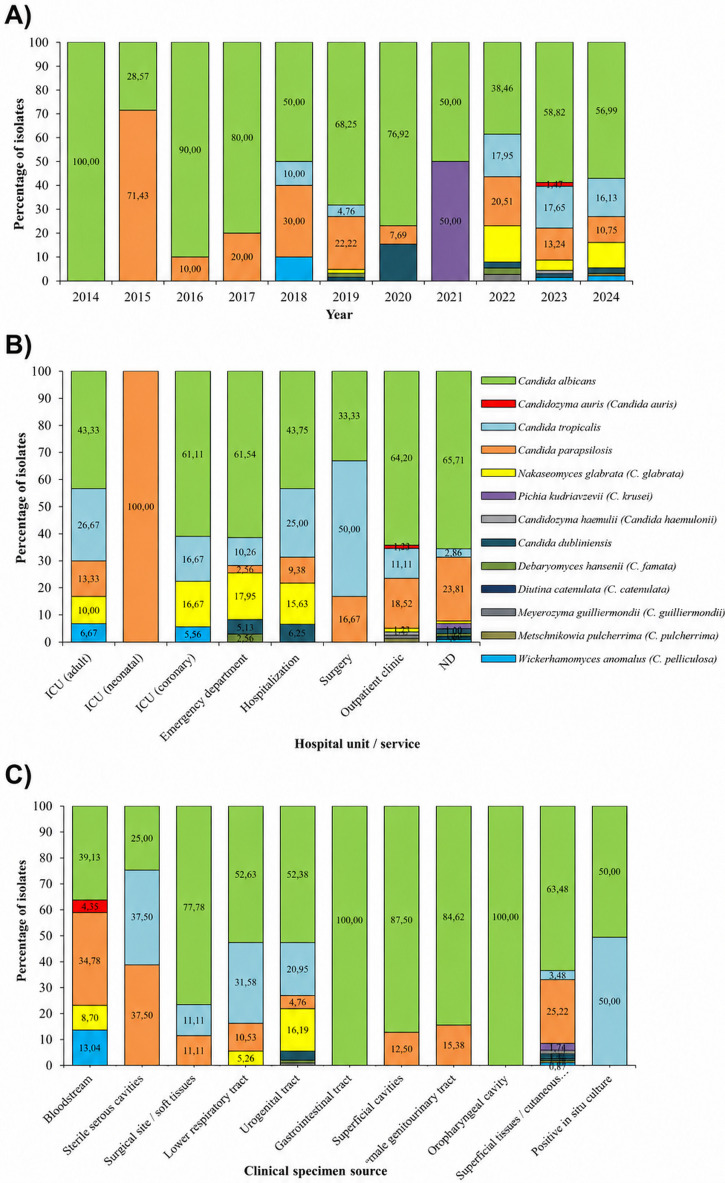
Proportional distribution of *Candida* spp. isolates (n = 314) according to A) year, B) hospital service/care setting, and C) clinical specimen source. Bars show category-specific percentages (100% stacked bars); the number of isolates (n) is indicated within each bar.

The time series showed a transition toward greater diversity, with non-*albicans* species proportions of approximately 41.2% (in 2023) and 43.0% (in 2024), and the detection of *C. auris* in 2023, highlighting the need for surveillance of emerging species (Fig 4A). By service, *C. albicans* predominated in outpatient and lower-complexity settings (outpatient clinic and emergency department), while non-*albicans* species were more prevalent in critical care areas. *C. parapsilosis* was reported as the only species in the neonatal ICU (n = 3) (Fig 4B).

Among confirmed isolates, *C. albicans* was the predominant species (58.92%; n = 185). Non-*albicans* species accounted for 41.08%, including *C. parapsilosis* (16.56%; n = 52), *C. tropicalis* (12.10%; n = 38), and *Nakaseomyces glabratus* (6.37%; n = 20). Less frequent species were detected, including *C. dubliniensis* (n = 6), *Wickerhamomyces anomalus* (n = 4), and *C. auris* (n = 1), in addition to other rare isolates.

The anatomical origin was concentrated in superficial tissues/cutaneous appendages (36.62%; n = 115) and urine (33.44%; n = 105). Blood samples constituted 7.32% (n = 23), followed by lower respiratory tract 6.05% (n = 19) and surgical site/soft tissue 5.73% (n = 18) (Fig 4C). These 23 microbiological blood-isolate records were not linked record by record to the 20 clinical/administrative candidemia records; therefore, both counts should be interpreted as complementary indicators from separate institutional information workflows rather than matched events. In the bloodstream subgroup (n = 23), the etiology was heterogeneous: *C. albicans* 39.13%, *C. parapsilosis* 34.78%, *W. anomalus* 13.04%, *N. glabratus* 8.70%, and *C. auris* 4.35%, highlighting the diversity in invasive origin (Fig 4C). Age also varied by species (*p* = 0.0168), with higher medians in species of greater clinical relevance (*C. albicans* 60 years; *C. tropicalis* 70 years; *N. glabratus* 68 years; *C. parapsilosis* 57 years).

### Associated factors (Multivariate models)

**Fig 5:**
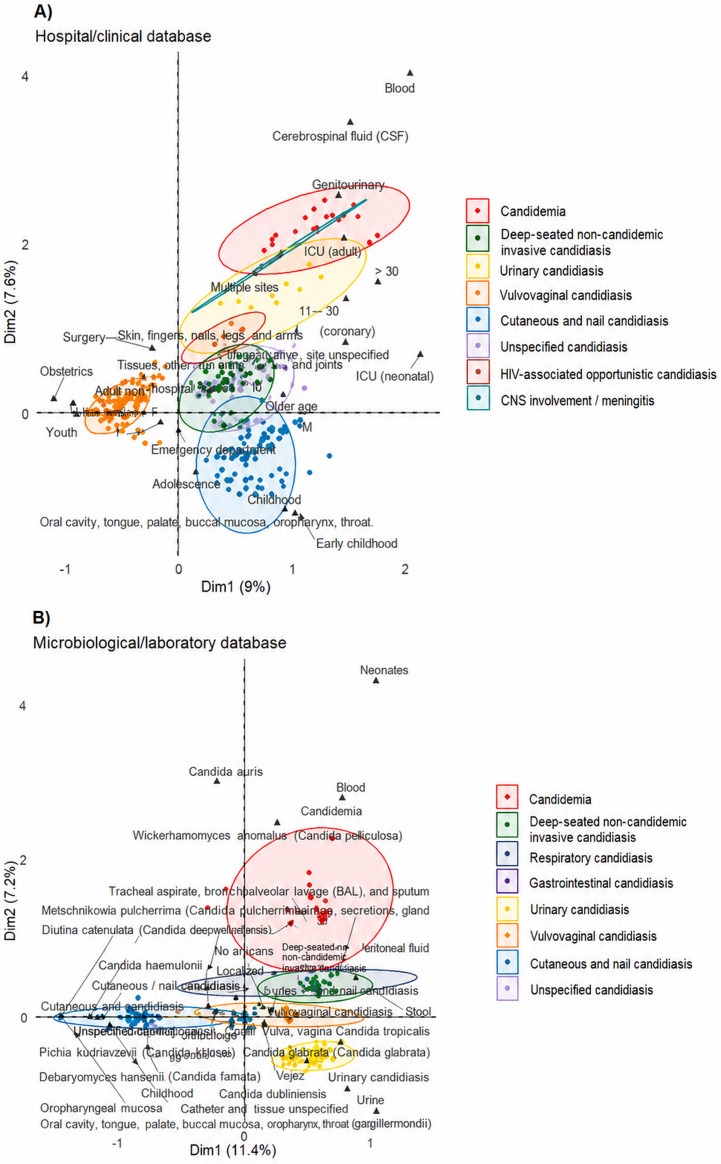
Multiple correspondence analysis (MCA) of *Candida* infection types according to data source. **A)** Hospital/clinical database, **B)** Microbiological/laboratory database**.**

Multiple correspondence analysis (MCA) was used as an exploratory approach to summarize co-occurrence patterns among categorical variables in the hospital/clinical database. The first two dimensions explained 9.1% (Dim1) and 7.7% (Dim2) of inertia (16.8% cumulative) and showed separation between invasive and mucocutaneous presentations: candidemia clustered with higher-complexity care, invasive procedures/devices, and blood samples, whereas mucocutaneous forms clustered with lower-complexity profiles (Fig 5A; S4 Table, S5 Table).

A second MCA based on microbiological isolate records incorporated clinical syndrome, sample origin, species, and available susceptibility interpretation categories. The first two dimensions explained 11.4% (Dim1) and 7.3% (Dim2) of inertia (18.7% cumulative). Candidemia clustered with blood samples and tracheal aspirate, while deep-seated non-candidemic invasive candidiasis clustered with sterile fluids; vulvovaginal candidiasis clustered mainly with *C. albicans*, *C. tropicalis* and *N. glabratus* and with vaginal/urinary samples (Fig 5B; S4 Table, S5 Table). Overall, MCA findings were interpreted descriptively and used to inform subsequent comparisons and regression modeling.

Exploratory multivariable models identified data-source-specific associations (Table 2). In the clinical/administrative database, clinically recorded candidemia showed an inverse association with short hospital stay (OR = 0.04; 95% CI: 0.01–0.14), whereas positive associations were observed with respiratory dysfunction (OR = 4.76; 95% CI: 1.23–16.39), invasive devices (OR = 5.54; 95% CI: 1.99–15.64), surgery (OR = 7.11; 95% CI: 1.201–30.87), and tumor (OR = 19.88; 95% CI: 3.13–97.51).

**Table 2:** Exploratory multivariable logistic regression models for candidemia, invasive candidiasis, and non-*albicans* isolation (vs *C. albicans*).

Variable	OR	IC 95%	*p*-value	Effect
**Candidemia**	–	–	<0.001	*p* value (LR test)
Short hospital stay	0.04	(0.01 - 0.14)	<0.001	Inverse association
Respiratory dysfunction	4.76	(1.23 - 16.39)	0.025	Associated factor
Invasive devices	5.54	(1.99 - 15.64)	0.001	Associated factor
Surgery	7.11	(1.20 - 30.87)	0.033	Associated factor
Tumor	19.88	(3.13 - 97.51)	0.003	Associated factor
**Invasive candidiasis**	–	–	<0.001	*p* value (LR test)
Age > 27 years	2.73	(1.61 - 4.85)	0.003	Associated factor
Male sex	4.98	(3.15 - 7.91)	<0.001	Associated factor
Cardiovascular disease	5.67	(1.41 - 18.91)	0.007	Associated factor
Tumor	9.51	(2.51 - 37.23)	0.0008	Associated factor
**Non-*albicans***	–	–	0.004	*p* value (LR test)
Candidemia	3.02	(1.04 - 9.23)	0.045	Associated factor
Age > 27 years	3.63	(1.39 - 11.48)	0.014	Associated factor
Urinary source	1.78	(1.04 - 3.04)	0.033	Associated factor

**Note**: Adjusted ORs (95% CI) from exploratory multivariable logistic regression models. For candidemia, Firth penalized logistic regression was used due to the low event frequency. For invasive candidiasis and non-*albicans* isolation, standard logistic regression was applied. Model-specific sample sizes were: candidemia model, n = 987, events = 20; invasive candidiasis model, n = 987, events = 101; non-*albicans* isolation model, n = 314, events = 129.

For invasive candidiasis, exploratory associations were observed with age > 27 years (OR = 2.73; 95% CI: 1.61–4.85), male sex (OR = 4.98; 95% CI: 3.15–7.91), cardiovascular disease (OR = 5.67; 95% CI: 1.41–18.91), and tumor (OR = 9.51; 95% CI: 2.51–37.23). In the microbiological isolate-level model, non-*albicans* isolation (vs *C. albicans*) showed associations with candidemia (OR = 3.02; 95% CI: 1.04–9.23), age > 27 years (OR = 3.63; 95% CI: 1.392–11.483), and urinary source (OR = 1.78; 95% CI: 1.04–3.04). These findings should be interpreted as exploratory associations within their respective data sources.

### Antifungal Susceptibility (Isolates with Available MIC)

Susceptibility testing was not available for all isolates; therefore, analyses were performed using isolates with recorded MICs, reporting denominators by species and antifungal agent (Table 3). In general, high activity of echinocandins (caspofungin and micafungin) was observed, with greater variability among azoles, particularly fluconazole. Five-fluorocytosine results were interpreted descriptively using EUCAST ECOFF-based WT/NWT categories and are reported separately in S6 Table to avoid confusion with clinical S/I/R interpretations. Species-specific denominators were uneven, and several species had fewer than 30 isolates; therefore, susceptibility percentages for low-frequency species should be interpreted descriptively as isolate-level surveillance signals rather than as representative institutional resistance rates or cumulative susceptibility estimates. No repeated de-identified medical-record codes were detected among the 196 AFST records. Because (Table 3) pools isolates from heterogeneous clinical sources, including superficial, urogenital, and bloodstream samples, these data should be viewed as a general microbiological survey and should not be used alone to guide empiric therapy for invasive candidiasis.

**Table 3:** Antifungal susceptibility profile of *Candida* species with available MICs according to clinically interpretable categories (S, SDD, I, R).

*Candida* spp.	Antifungal agent	S n (%)	SDD n (%)	I n (%)	R n (%)	Total n (%)
*Candida albicans*	Fluconazole	96(89.71)	3(2.80)	–	8(7.47)	107(100)
	Voriconazole	103(96.27)	–	–	4(3.73)	107(100)
	Caspofungin	102(95.33)	–	3(2.80)	2(1.87)	107(100)
	Micafungin	100(93.46)	–	3(2.80)	4(3.74)	107(100)
*Candida tropicalis*	Fluconazole	33(97.05)	–	–	1(2.95)	34(100)
	Voriconazole	33(97.05)	–	–	1(2.95)	34(100)
	Caspofungin	34(100)	–	–	–	34(100)
	Micafungin	34(100)	–	–	–	34(100)
*Candida parapsilosis*	Fluconazole	23(85.19)	1(3.70)	–	3(11.11)	27(100)
	Voriconazole	24(88.90)	–	2(7.40)	1(3.70)	27(100)
	Caspofungin	26(96.30)	–	1(3.70)	–	27(100)
	Micafungin	27(100)	–	–	–	27(100)
*Nakaseomyces glabratus*	Fluconazole	–	–	–	–	–
*(C. glabrata)*	Voriconazole	13(68.42)	–	6(31.57)	–	19(100)
	Caspofungin	–	–	–	–	–
	Micafungin	18(94.74)	–	–	1(5.26)	19(100)
*Candidozyma haemuli*	Fluconazole	–	–	–	1(100)	1(100)
*(Candida haemulonii)*	Voriconazole	1(100)	–	–	–	1(100)
	Caspofungin	–	–	1(100)	–	1(100)
	Micafungin	–	–	–	–	–
*Candida dubliniensis*	Fluconazole	1(25.00)	–	–	3(75.00)	4(100)
	Voriconazole	3(75.00)	–	–	1(25.00)	4(100)
	Caspofungin	3(100)	–	–	–	3(100)
	Micafungin	–	–	–	–	–
*Meyerozyma guilliermondii*	Fluconazole	1(100)	–	–	–	1(100)
*(C. guilliermondii)*	Voriconazole	1(100)	–	–	–	1(100)
	Caspofungin	1(100)	–	–	–	1(100)
	Micafungin	1(100)	–	–	–	1(100)
*Wickerhamomyces anomalus*	Fluconazole	3(100)	–	–	–	3(100)
*(C. pelliculosa)*	Voriconazole	–	–	2(66.66)	1(33.33)	3(100)
	Caspofungin	2(66.66)	–	–	1(33.33)	3(100)
	Micafungin	–	–	–	–	–

**Note:** S, susceptible; SDD, susceptible dose-dependent; I, intermediate; R, resistant. 5-FC results are reported separately in Table S6. Species with fewer than 30 isolates are reported descriptively and should be interpreted as isolate-level surveillance signals rather than representative institutional resistance rates or cumulative susceptibility estimates. AFST analyses were performed at the isolate-record level; no repeated de-identified medical-record codes were detected among the 196 AFST records. Because this table pools isolates from heterogeneous clinical sources, including superficial, urogenital, and bloodstream samples, it should be interpreted as a general microbiological survey and not as a standalone guide for empiric therapy in invasive candidiasis.

In *C. albicans* (n = 107), fluconazole showed 89.71% susceptibility, with 7.47% resistance, and voriconazole 96.27% susceptibility; caspofungin and micafungin showed >93% susceptibility. In *C. tropicalis* (n = 34), fluconazole and voriconazole reached 97.05% susceptibility, and echinocandins 100%. In *C. parapsilosis* (n = 27), fluconazole showed lower performance (S 85.19%; R 11.11%) compared to echinocandins, and micafungin remained 100% susceptible. In *N. glabratus* (n = 19), a more limited profile was observed, with preserved susceptibility to micafungin (94.74%) and a higher proportion in the intermediate category for voriconazole.

## Discussion

In this retrospective study (2014–2024), the recorded candidiasis burden was 0.41% (4.06 cases per 1000 institutional care records). Although low in absolute terms, this represents a sustained burden for a high-complexity hospital and is consistent with ranges reported in Latin American institutions with comparable care profiles [1,12,13]. In Colombia, reference hospital series have described similar magnitudes, suggesting that this behavior is not isolated and providing a baseline for a region with historically limited evidence [4,14]. The main contribution of this work is the longitudinal characterization and the combined clinical–microbiological perspective to support local surveillance and decision-making.

The low recorded candidemia rate should be interpreted cautiously. Candidemia represented a small subset of the overall *Candida* infection burden captured in this study, whereas the main analysis included all clinically recorded candidiasis forms. Because candidemia identification in the clinical/administrative database depended on retrospective diagnostic coding and was not linked record by record to laboratory-confirmed bloodstream isolates, the recorded rate may underestimate the true invasive disease burden. Consequently, the candidemia model should be interpreted as an exploratory analysis based on clinically recorded administrative data rather than as a definitive patient-level clinical–microbiological inference.

The temporal pattern showed increasing diagnoses up to 2018 followed by a decline. During the 2018–2019 period, the hospital did not change its information software, and no shift from ICD-9 to ICD-10 coding occurred. Therefore, this pattern should not be attributed to a documented change in the Hospital Information System or coding system. However, as a record-based retrospective analysis, trends should be interpreted cautiously because changes in clinical awareness, diagnostic capacity, sampling practices, coding intensity, infection control measures, or antimicrobial optimization initiatives may influence case capture without necessarily reflecting true changes in incidence [15–17]. The key finding is the persistence of candidiasis across the decade, with cumulative burden over time.

From a microbiological perspective, Candida spp. comprised 40.46% of fungal isolates, consistent with clinical laboratory reports where Candida constitutes a substantial fraction of isolated fungi and remains a leading agent of healthcare-associated mycoses [18–20]. The gap between clinical diagnoses and laboratory-confirmed isolates is expected, as many mucocutaneous infections are diagnosed and treated empirically without systematic microbiological confirmation [21,22]. Rather than a limitation, this highlights the need to standardize clinical definitions and to prioritize microbiological confirmation when management is likely to change (invasive disease, recurrences, or treatment failure).

The 2014–2024 period also captures a transition in institutional microbiological capacity. Earlier years relied mainly on routine clinical and laboratory records with more limited microbiological granularity, whereas later years incorporated automated identification and susceptibility testing and structured WHONET MIC exports. This transition likely improved species-level identification, MIC availability, data standardization, and detection of less frequent or emerging species, supporting the value of the longitudinal design while reinforcing the need for cautious interpretation of comparisons across years.

Clinically, mucocutaneous disease predominated, particularly vulvovaginal and oropharyngeal candidiasis, consistent with global epidemiology and the expected burden in women of reproductive age [23,24]. The relatively large other/unspecified category represents an important limitation of this retrospective record-based study. It reflects limited diagnostic granularity in clinical/administrative coding and may have led to misclassification or under-recognition of more specific clinical forms, particularly when the anatomical site or invasive status was not clearly documented. Although invasive forms were less frequent, their impact is disproportionate: candidemia and deep-seated invasive candidiasis are associated with higher severity, longer hospital stays, and increased resource use in international and regional cohorts [25,26]. The longer stays observed in candidemia and deep-seated non-candidemic invasive candidiasis, including CNS involvement/meningitis, align with this clinical pattern and support reporting outcomes separately for mucocutaneous/non-invasive versus invasive presentations [27]. For oropharyngeal candidiasis, concentration in early childhood and in those aged ≥60 years may indicate vulnerability and frailty across services rather than only classic severe immunosuppression [28].

In terms of species distribution, C. albicans remained the predominant species, which is consistent with its recognized role as a major etiologic agent of mucocutaneous candidiasis and hospital-associated Candida infections. This predominance was expected in our dataset because the clinical spectrum was dominated by mucocutaneous disease, particularly vulvovaginal and oropharyngeal candidiasis. However, the proportion of non-albicans Candida species was substantial, mainly represented by C. parapsilosis, C. tropicalis, and N. glabratus. This finding is consistent with previous studies describing an epidemiological shift toward non-albicans Candida species, especially in healthcare-associated contexts, where antimicrobial exposure, invasive devices, critical care, and improved laboratory identification may favor the detection of species with different ecological and susceptibility profiles [29,30–34]. In our study, the predominance of C. albicans in outpatient and lower-complexity settings contrasted with the greater representation of non-albicans Candida species in critical care areas, supporting the interpretation that species distribution varies according to clinical context and specimen source.

Detection of emerging species in invasive samples, although infrequent, is a relevant surveillance signal. The identification of C. auris—even as a single recent isolate—aligns with regional alerts regarding spread and nosocomial transmission risk, particularly in critical care settings [35–37]. Likewise, the presence of W. anomalus in blood is consistent with reports of healthcare-associated infection and potential nosocomial events [36].

The multivariable analyses should be interpreted as exploratory and data-source specific, because the clinical/administrative and microbiological databases were not linked record by record. For clinically recorded candidemia, invasive devices, surgery, tumors, and respiratory dysfunction showed associations within the clinical/administrative database, consistent with clinical patterns described in critically ill post-surgical patients with organ dysfunction [38–41]. For clinically recorded invasive candidiasis, associations with male sex, older age, and cardiovascular comorbidity are plausible and align with cohorts in which comorbidity burden and hospital exposure influence severity and clinical outcomes [42,43]. In the microbiological isolate-level model, non-*albicans* isolation showed associations with candidemia, adulthood, and urinary origin, suggesting that higher-complexity scenarios and selected anatomical foci may concentrate species with less predictable susceptibility profiles [43–46].

The inverse association between short hospital stay and clinically recorded candidemia should also be interpreted cautiously and not as a protective effect. This finding likely reflects the case mix of the clinical/administrative database, in which most episodes corresponded to mucocutaneous/non-invasive candidiasis, particularly vulvovaginal candidiasis, which is commonly managed in lower-complexity or short-stay settings.

Antifungal susceptibility findings should be interpreted according to the clinical source of the isolates. Because most isolates were obtained from mucocutaneous, superficial, or urogenital samples rather than invasive specimens, the pooled susceptibility results should be considered primarily as descriptive microbiological surveillance data. In mucocutaneous and urogenital candidiasis, these findings help describe species-level susceptibility patterns and azole variability, particularly the lower fluconazole performance observed in C. parapsilosis and the more limited profile observed in N. glabratus [47,48]. For invasive candidiasis, interpretation should be more cautious because the bloodstream subgroup was small and species-specific denominators were limited. Although echinocandin activity was high among predominant species and is consistent with international surveillance [29,27], these pooled data should not be used alone to guide empiric therapy for invasive candidiasis or to define institutional treatment protocols. Susceptibility percentages for species represented by fewer than 30 isolates should also be interpreted cautiously, because one or two isolates can substantially change the estimated proportion. Therefore, results for low-frequency or emerging species such as C. auris, C. dubliniensis, N. glabratus, and W. anomalus should be considered isolate-level surveillance signals rather than representative institutional resistance rates. Overall, these findings support species-level identification and isolate-level susceptibility testing in clinically significant infections, while avoiding overstatement of clinical implications [49].

The 5-fluorocytosine pattern, with predominance of non-WT classifications, should be interpreted cautiously. As EUCAST recommends ECOFF-based WT/NWT interpretation and NWT does not necessarily imply clinical failure, this may reflect surveillance artifacts related to cut-off availability, system configuration, or routine reporting rather than a direct therapeutic conclusion [50]. Given that uniformly high NWT proportions are not typically expected in multicenter studies, this finding warrants targeted verification of the reporting workflow.

Overall, candidiasis in this hospital appears to involve two scenarios: a frequent, mainly outpatient mucocutaneous burden and a less frequent but clinically critical invasive burden characterized by greater etiological diversity, emerging species signals, and exploratory clinical associations. These data provide a descriptive surveillance baseline for strengthening local monitoring, microbiological confirmation, and antifungal stewardship discussions in high-complexity care settings.

### Conclusions

This study provides a longitudinal clinical–microbiological survey of *Candida* infections in a single high-complexity hospital in Tolima, Colombia, during 2014–2024. The findings describe a low but sustained recorded burden of candidiasis, with predominance of *C. albicans* and a substantial proportion of non-*albicans* species, mainly *C. parapsilosis* and *C. tropicalis*. Most cases were mucocutaneous, whereas recorded invasive forms were less frequent and should be interpreted cautiously because of the low number of recorded candidemia cases and the use of separate, non-linked clinical/administrative and microbiological data sources.

The susceptibility findings suggest high echinocandin activity among predominant species and variability among some non-*albicans* isolates. However, because susceptibility data were pooled from heterogeneous clinical specimens and several species were represented by small denominators, these results should be interpreted as descriptive isolate-level surveillance signals rather than as a standalone basis for empiric therapy or institutional treatment protocols. Overall, the study provides a local surveillance baseline that supports the need for species-level identification, isolate-level susceptibility testing, and improved microbiological confirmation in clinically significant *Candida* infections.

## Supporting information

S1 Checklist STROBE and RECORD. (DOCX)

S1 Text Supplementary Methods S1. Data sources and processing flow.
(DOCX)

S2 Text Supplementary Methods S2. Antifungal susceptibility interpretation.
(DOCX)

S3 Text Supplementary Methods S3. Multiple correspondence analysis (MCA).
(DOCX)

S1 Table ICD-10 codes used to define candidiasis cases in the hospital database (2014–2024). (DOCX)

S2 Table Breakpoints/ECOFF sources and interpretation rules (CLSI/EUCAST). (DOCX)

S3 Table Raw WHONET MIC records and antifungal interpretation categories for *Candida* spp. isolates, 2022–2024. (DOCX)

S4 Table Variables and coding used in multiple correspondence analysis (MCA). (DOCX)

S5 Table Top contributing categories to MCA dimensions. (DOCX)

S6 Table Descriptive 5-fluorocytosine profile according to ECOFF-based WT/NWT categories. (DOCX)
