# Supplementary material for: Clinical and microbiological epidemiology of Candida infections in a high-complexity hospital in Tolima, Colombia (2014–2024)
Source: PLoS One. 2026 Jul 24;21(7):e0354684. doi: 10.1371/journal.pone.0354684 (PMC13399354; doi:10.1371/journal.pone.0354684)
Supplement: S1 Checklist — (DOCX) [file pone.0354684.s001.docx]

**S1 Checklist.** STROBE and RECORD

checklist mapping for the revised manuscript, Strobe.

| **STROBE item** | **What to report** | **Where in manuscript** | **Page/Line** | **Notes/NA** |
| --- | --- | --- | --- | --- |
| 1a | Design indicated in title/abstract | Abstract (Methods) | p2 L23–29 | “retrospective observational study”. |
| 1b | Informative, balanced abstract | Abstract | p2 L19–38; p3 L39–43 | OK. |
| 2 | Background/rationale | Introduction | p3 L44–64 | OK. |
| 3 | Objectives | Introduction (objective) | p4 L65–70 | OK. |
| 4 | Study design (key elements early) | Methodology – Design and setting | p4 L72–82 | OK. |
| 5 | Setting, location, dates | Methods + study period + MIC period | p4 L74–82; p5 L83–90 | OK (2014–2024; MIC 2022–2024). |
| 6a | Eligibility criteria + selection methods | Population/definitions/selection | p5 L93–106 | OK. |
| 6b | Follow-up / matching (as applicable) | Not applicable | NA | **NA** (no follow-up/matching design). |
| 7 | Define outcomes/exposures/predictors/confounders | Methods + regression outcomes | p5 L99–106; p6–7 L120–134; p14–15 L243–255 | Partial: outcomes/predictors are described in Methods and Table 2. |
| 8 | Data sources/measurement | Methods (sources; laboratory; AST) | p4 L74–82; p5 L83–90; p6 L107–119 | OK. |
| 9 | Bias (efforts to address) | Discussion (trend caution; routine-data limitations; 5-FC artifacts) | p18–19 L297–320; p20 L337–343; p22 L379–394 | Partial. |
| 10 | Study size | Results denominators | p8 L148–157 | OK (n=987 episodes; n=776 isolates; n=314 Candida). |
| 11 | Handling quantitative variables | Statistical analysis | p6–7 L120–129 | OK (age/LOS categorized; median/IQR). |
| 12a | Statistical methods incl confounding | Statistical analysis | p6–7 L120–134 | OK (logistic regression; Firth for candidemia; complete-case analysis). |
| 12b | Subgroups/interactions | Results by syndrome/service/species; MIC subset | p8–18 L148–295 | Subgroups described; interactions not reported (OK). |
| 12c | Missing data handling | Statistical analysis + MIC denominators | p7 L129–134; p15 L265–278; Table 3 p16–18 | OK (complete-case analysis; denominators by species/antifungal for MIC). |
| 12d | Loss to follow-up / matching | Not applicable | NA | NA. |
| 12e | Sensitivity analyses | Not reported | — | **NA** (no sensitivity analyses described). |
| 13a | Numbers at each stage | S1 Text (selection-stage counts) referenced in Methods | p5 L88–92 (see S1 Text) | In S1 Text (selection-stage counts). |
| 13b | Reasons for non-participation | S1 Text | p5 L96–98 (see S1 Text) | In S1 Text (duplicates; no corroboration; missing key variables). |
| 13c | Flow diagram | Not included | — | NA (text-only counts in S1 Text; no flow chart). |
| 14a | Participant characteristics | Results + Table 1 | p9–12 L176–197 | OK. |
| 14b | Missing data per variable | Partly via MIC availability statement and susceptibility tables | p2 L37–38; p15 L265–278; Table 3 p16–18; S3 Table | Partial: MIC availability is explicitly reported; overall missingness is not tabulated. |
| 14c | Follow-up time | Not applicable | — | **NA**. |
| 15 | Outcome data | Results (forms; candidemia; species; MIC) | p8–18 L148–295 | OK. |
| 16a | Main results (adjusted estimates + precision) | Results + Table 2 | p14–15 L243–255; Table 2 p15–16 | OK. |
| 16b | Category boundaries for categorized variables | Statistical analysis | p7 L128–129 | OK. |
| 16c | Absolute risk translation | Not applicable | — | **NA**. |
| 17 | Other analyses | MCA + MIC analyses | p14 L227–241; p15–18 L265–295 | OK (MCA described; MIC subset). |
| 18 | Key results summarized | Discussion + Conclusions | p18–23 L297–416 | OK. |
| 19 | Limitations | Discuss limitations/bias/imprecision | p18–22 L306–400 | OK. |
| 20 | Interpretation | Discussion | p18–22 L297–400 | OK. |
| 21 | Generalisability | Discussion + Conclusions | p20 L337–343; p22 L389–400; p23 L401–416 | As a single-hospital study, findings reflect institutional burden and may not be generalizable to population incidence. |
| 22 | Funding | Acknowledgments/Funding statement | p23–24 L418–424 | OK (scholarship support acknowledged; no funding for study conduct/data collection/analysis). |

**B.** RECORD (routinely collected health data) – mapping

| **RECORD item** | **What to report** | **Where in manuscript** | **Page/Line** | **Notes/NA** |
| --- | --- | --- | --- | --- |
| 1.1 | Type of data (routinely collected) | Abstract/Methods | p2 L23–29; p4 L74–82 | OK (administrative/clinical records + laboratory database). |
| 1.2 | Name of databases | Abstract/Methods | p2 L23–25; p4 L75–78; p5 L83–90 | OK (microbiology laboratory database/WHONET mentioned). |
| 1.3 | Geographic region + timeframe | Title/Abstract/Methods | p1 L3–4; p2 L23–24; p4 L74–75 | OK. |
| 6.1 | Population selection incl codes/algorithms | Methods + ICD-10 table | p5 L93–106 (see S1 Table) | OK (ICD-10 codes in S1 Table). |
| 6.2 | Validation of codes/algorithms | Not reported | — | NA/Not done (no validation study cited). |
| 6.3 | Linkage (if used) | Methods | p4 L75–80; p7 L131–134 | OK (no record-by-record linkage performed; stated). |
| 7.1 | Codes/algorithms for exposures/outcomes/confounders | ICD-10 + MCA coding + regression variables | p5 L99–106; p6–7 L120–134 (see S1 Table, S4 Table and S5 Table) | Partly in Supporting Information (S1 Table, S4 Table and S5 Table). |
| 7.2 | Validity/accuracy of codes/algorithms | Discussion/limitations of routine data | p18–20 L306–343; p22 L379–394 | Partial: coding validity/accuracy is addressed as a routine-data limitation; no separate validation study was performed. |
| 8.1 | Database description: setting/coverage/collection | Methods | p4 L74–82; p5 L83–90; p6 L107–119 | OK. |
| 12.1 | Extent of access to database population | S1 Text referenced in Methods | p5 L88–92 (see S1 Text) | In S1 Text (extent of access described there). |
| 12.2 | Data cleaning methods | Duplicates/exclusions; MIC filtering | p5 L96–98; p6 L118–119; p15 L265–278; S3 Table | OK. |
| 12.3 | Linkage statement and evaluation | Methods | p4 L75–80; p7 L131–134 | OK (no linkage performed). |
| 13.1 | Selection of persons incl filtering by data quality | Methods (exclusions/filtering) | p5 L93–106; p6 L118–119 | OK. |
| 13.2 | Linkage numbers | Not applicable | — | NA (no linkage). |
| 13.3 | Flow diagram | Not included | — | NA. |
| 14.1 | Extent of missing data | Missing handling + MIC denominators | p7 L129–134; p2 L37–38; p15 L265–278; Table 3 p16–18; S3 Table | Partial: MIC availability explicitly reported; overall missingness not tabulated. |
| 15.1 | Outcome events using routine definitions | Results | p8–18 L148–295 | OK. |
| 16.1 | Variable handling/adjustment | Methods + Table 2 | p6–7 L120–134; p14–15 L243–255; Table 2 p15–16 | OK. |
| 17.1 | Other analyses | MCA + MIC analyses | p14 L227–241; p15–18 L265–295 | OK. |
| 19.1 | Limitations of routine data | Discussion (record-based trend caution; reporting artifacts) | p18–22 L306–400 | Partial: underreporting/coding variability/colonization vs infection/single-center limitations are discussed. |
| 22.1 | Access to protocol/raw data/code | Data Availability statement | p24–25 L436–445 | OK (Zenodo DOI, GitHub repository, de-identified datasets/code, and restrictions on original institutional records). |
