# Supplementary material for: Clinical and microbiological epidemiology of Candida infections in a high-complexity hospital in Tolima, Colombia (2014–2024)
Source: PLoS One. 2026 Jul 24;21(7):e0354684. doi: 10.1371/journal.pone.0354684 (PMC13399354; doi:10.1371/journal.pone.0354684)
Supplement: S1 Text — Data sources and processing flow. (DOCX) [file pone.0354684.s002.docx]

**S1 Text. Supplementary Methods S1.** Data sources and processing flow.

The study used two institutional sources analysed in parallel: (A) the hospital administrative/clinical database (2014–2024), analyzed at the level of patient infection episodes/records, and (B) the Microbiology Laboratory database (2014–2024), analyzed at the level of microbiological isolate records (each isolate corresponds to a patient sample). No cross-database linkage was performed at the individual-patient level.

Laboratory identification of Candida spp. was based on routine practice across the study period (conventional culture and/or BD Phoenix in 2014–2021; VITEK 2 Compact in 2022–2024). Structured MIC-based antifungal susceptibility data were only available for 2022–2024, when MIC values were routinely captured and exported in WHONET format; therefore, MIC-based susceptibility analyses were restricted to 2022–2024, while clinical epidemiology and species distribution analyses used the full 2014–2024 period.

WHONET-format exports for 2022–2024 were obtained directly from the Microbiology Laboratory and filtered to retain Candida spp. isolate records only. MIC availability varied by species and routine testing panel; isolates without MIC results for a given antifungal were treated as “not tested/not reported” and excluded from that antifungal-specific analysis. MIC availability denominators are reported in Supplementary Table 3, and ICD-10 coding used to define mucocutaneous candidiasis in the hospital database is provided in Supplementary Table 1.
