# Supplementary material for: Clinical and microbiological epidemiology of Candida infections in a high-complexity hospital in Tolima, Colombia (2014–2024)
Source: PLoS One. 2026 Jul 24;21(7):e0354684. doi: 10.1371/journal.pone.0354684 (PMC13399354; doi:10.1371/journal.pone.0354684)
Supplement: S2 Text — Antifungal susceptibility interpretation. (DOCX) [file pone.0354684.s003.docx]

**Supplementary Methods S2.** Antifungal susceptibility interpretation

MICs (µg/mL) obtained from routine testing (VITEK 2 Compact; WHONET-format exports, 2022–2024) were interpreted using current CLSI guidance (S/I/R) when clinical breakpoints were available. Because clinical breakpoints are not available for 5-flucytosine (5-FC) for several *Candida* species, 5-FC was analysed exclusively using EUCAST ECOFF-based classification and reported as wild-type (WT) or non-wild-type (NWT), following the interpretation approach described in [11]. Species-specific WT thresholds applied in this study were those summarized in [11] and are detailed in Supplementary Table 2. Denominators and MIC availability by antifungal and species are reported in Supplementary Table 3, and ICD-10 coding used to define mucocutaneous syndromes is provided in Supplementary Table 1.
