# Supplementary material for: Clinical and microbiological epidemiology of Candida infections in a high-complexity hospital in Tolima, Colombia (2014–2024)
Source: PLoS One. 2026 Jul 24;21(7):e0354684. doi: 10.1371/journal.pone.0354684 (PMC13399354; doi:10.1371/journal.pone.0354684)
Supplement: S3 Text — Multiple correspondence analysis (MCA). (DOCX) [file pone.0354684.s004.docx]

**S3 Text. Supplementary Methods S3.** Multiple correspondence analysis (MCA).

MCA was used as an exploratory approach to summarize co-occurrence patterns among categorical variables and to support descriptive interpretation of clinical and microbiological profiles. Two separate MCAs were conducted: (i) a clinical MCA using the hospital administrative/clinical database (episode-level records) and (ii) a laboratory MCA using microbiological isolate records. Variables included those reported in the main text and tables (clinical form/syndrome, sex, age group, service/area of care, sample origin, socioeconomic stratum, and selected clinical/procedural markers where available). Missing/unknown values were retained as an explicit category (e.g., “ND/Unknown”) when present in the source data. To reduce sparse-category instability, categories with very low frequency were grouped when applicable, and only categories with sufficient counts were interpreted. MCA outputs (inertia explained by dimensions, category contributions, and cos²) were used descriptively to characterize the separation of clinical presentations and to guide subsequent comparisons and regression modeling; no inferential claims were derived from MCA alone. Dimension-specific summaries (top contributing categories and cos²) are reported in Supplementary Table 5, and variable coding is detailed in Supplementary Table 4.
