# Supplementary material for: Clinical and microbiological epidemiology of Candida infections in a high-complexity hospital in Tolima, Colombia (2014–2024)
Source: PLoS One. 2026 Jul 24;21(7):e0354684. doi: 10.1371/journal.pone.0354684 (PMC13399354; doi:10.1371/journal.pone.0354684)
Supplement: S1 Table — (DOCX) [file pone.0354684.s005.docx]

**Supplementary. S1 Table .**  ICD-10 codes used to define candidiasis cases in the hospital database (2014–2024).

| **ICD-10 Code** | **Diagnosis** | **n** |
| --- | --- | --- |
| B20.4 | HIV disease resulting in candidiasis | 8 |
| B37.0 | Candidal stomatitis | 240 |
| B37.2 | Candidiasis of skin and nails | 10 |
| B37.3 | Candidiasis of vulva and vagina | 500 |
| B37.4 | Candidiasis of other urogenital sites | 10 |
| B37.7 | Candidal septicemia | 20 |
| B37.8 | Candidiasis of other sites | 77 |
| B37.9 | Candidiasis, unspecified | 118 |
| G02.1 | Meningitis in mycoses | 4 |
| **Total** |  | **987** |

**Note:** Note: ICD-10 codes correspond to institutional diagnostic coding used for case identification in the hospital database. B37.8 and G02.1 were operationally reviewed within the deep-seated non-candidemic invasive candidiasis category when candidemia was not recorded.
