# Supplementary material for: Clinical and microbiological epidemiology of Candida infections in a high-complexity hospital in Tolima, Colombia (2014–2024)
Source: PLoS One. 2026 Jul 24;21(7):e0354684. doi: 10.1371/journal.pone.0354684 (PMC13399354; doi:10.1371/journal.pone.0354684)
Supplement: S2 Table — (DOCX) [file pone.0354684.s006.docx]

**Supplementary. S2 Table.** Breakpoints/ECOFF sources and interpretation rules (CLSI 2017/2022; EUCAST. 2025; Arendrup. 2024).

| **Species** | **Antifungal agent** | **Clinical breakpoints (μg/mL)** | | | |  |  |
| --- | --- | --- | --- | --- | --- | --- | --- |
|  |  | **S /** | **SDD** | **I** | **R /** | **WT*** | **nWT*** |
| *C. albicans* | Fluconazole | ≤2 | 4 | - | ≥8 |  |  |
|  | Voriconazole | ≤0.12 | - | 0.25 – 0.5 | ≥1 |  |  |
|  | Anidulafungin | ≤0.25 | - | 0.5 | ≥1 |  |  |
|  | Caspofungin | ≤0.25 | - | 0.5 | ≥1 |  |  |
|  | Micafungin | ≤0.25 | - | 0.5 | ≥1 |  |  |
|  | Amphotericin B | ≤1 |  |  | ≥1 |  |  |
|  | 5-Fluorocytosine* |  |  |  |  | ≤0.5 | ≥0.5 |
| *C. glabrata* | Fluconazole | - | ≤32 | - | ≥64 |  |  |
| *(Nakaseomyces glabratus)* | Voriconazole | - | - | - | - |  |  |
|  | Anidulafungin | ≤0.12 | - | 0.25 | ≥0.5 |  |  |
|  | Caspofungin | ≤0.12 | - | 0.25 | ≥0.5 |  |  |
|  | Micafungin | ≤0.06 | - | 0.12 | ≥0.25 |  |  |
|  | Amphotericin B | ≤2 |  |  | ≥2 |  |  |
|  | 5-Fluorocytosine* |  |  |  |  | ≤0.5 | ≥0.5 |
| *C. guilliermondii* | Fluconazole | - | - | - | - |  |  |
| (*Meyerozyma guilliermondii*) | Voriconazole | - | - | - | - |  |  |
|  | Anidulafungin | ≤2 | - | 4 | ≤8 |  |  |
|  | Caspofungin | ≤2 | - | 4 | ≤8 |  |  |
|  | Micafungin | ≤2 | - | 4 | ≤8 |  |  |
|  | Amphotericin B | ≤1 | - | - | ≥1 |  |  |
|  | 5-Fluorocytosine* |  |  |  |  | ≤1 | ≥1 |
| *C. krusei* | Fluconazole | - | - | - | - |  |  |
| *(Pichia kudriavzevii)* | Voriconazole | ≤0.5 | - | 1 | ≥2 |  |  |
|  | Anidulafungin | ≤0.25 | - | 0.5 | ≥1 |  |  |
|  | Caspofungin | ≤0.25 | - | 0.5 | ≥1 |  |  |
|  | Micafungin | ≤0.25 | - | 0.5 | ≥1 |  |  |
|  | Amphotericin B | ≤1 | - | - | ≥1 |  |  |
|  | 5-Fluorocytosine* |  |  |  |  | ≤8 | ≥8 |
| *C. parapsilosis* | Fluconazole | ≤2 | 4 | - | ≥8 |  |  |
|  | Voriconazole | ≤0.12 | - | 0.25 – 0.5 | ≥1 |  |  |
|  | Anidulafungin | ≤2 | - | 4 | ≥8 |  |  |
|  | Caspofungin | ≤2 | - | 4 | ≥8 |  |  |
|  | Micafungin | ≤2 | - | 4 | ≥8 |  |  |
|  | Amphotericin B | ≤1 | - | - | ≥1 |  |  |
|  | 5-Fluorocytosine* |  |  |  |  | ≤0.5 | ≥0.5 |
| *C. tropicalis* | Fluconazole | ≤2 | 4 | - | ≥8 |  |  |
|  | Voriconazole | ≤0.12 | - | 0.25 – 0.5 | ≥1 |  |  |
|  | Anidulafungin | ≤0.25 | - | 0.5 | ≥1 |  |  |
|  | Caspofungin | ≤0.25 | - | 0.5 | ≥1 |  |  |
|  | Micafungin | ≤0.25 | - | 0.5 | ≥1 |  |  |
|  | Amphotericin B | ≤1 | - | - | ≥1 |  |  |
|  | 5-Fluorocytosine* |  |  |  |  | ≤0.5 | ≥0.5 |

**Note: abbreviations: S. susceptible; SDD. susceptible dose-dependent; I. intermediate; R. resistant.**

**(Wild-type and non-wild-type)* only for 5-fluorocytosine***
