# Supplementary material for: Clinical and microbiological epidemiology of Candida infections in a high-complexity hospital in Tolima, Colombia (2014–2024)
Source: PLoS One. 2026 Jul 24;21(7):e0354684. doi: 10.1371/journal.pone.0354684 (PMC13399354; doi:10.1371/journal.pone.0354684)
Supplement: S3 Table — (DOCX) [file pone.0354684.s007.docx]

**Supplementary. S3 Table.** Raw WHONET MIC records and antifungal interpretation categories for *Candida* spp. isolates, 2022–2024.

| **SPECIES *Candida* spp.** | **Year** | **FCT_NM** | **CAS_NM** | **FLU_NM** | **MIF_NM** | **VOR_NM** | **Amphotericin B (AB_NM)** | **5-Fluorocytosine (FCT_NM)** | **Caspofungin (CAS_NM)** | **Fluconazole (FLU_NM)** | **Micafungin (MIF_NM)** | **Voriconazole (VOR_NM)** |
| --- | --- | --- | --- | --- | --- | --- | --- | --- | --- | --- | --- | --- |
| *Candida albicans* | 2022 | <=1 | <=.12 | <=.5 | <=.06 | <=.12 | ND | NWT | S | S | S | S |
| *Nakaseomyces glabratus (Candida glabrata)* | 2022 | <=1 |  |  | <=.06 | .25 | ND | NWT | ND | ND | R | I |
| *Candida tropicalis* | 2022 | <=1 | <=.12 | 1 | <=.06 | <=.12 | ND | NWT | S | S | S | S |
| *Meyerozyma guilliermondii (Candida guilliermondii)* | 2022 | <=1 | <=.12 | 1 | <=.06 | <=.12 | ND | WT | S | S | S | S |
| *Candida albicans* | 2022 | >32 | >4 | 8 | >4 | <=.12 | ND | NWT | R | R | R | S |
| *Candida dubliniensis* | 2022 | >32 |  | 8 |  | >4 | ND | NWT | ND | R | ND | R |
| *Candida tropicalis* | 2022 | <=1 | .25 | <=.5 | <=.06 | <=.12 | ND | NWT | S | S | S | S |
| *Candida parapsilosis* | 2022 | <=1 | .5 | <=.5 | .5 | <=.12 | ND | NWT | S | S | S | S |
| *Candida albicans* | 2022 | <=.5 | .5 | <=.5 | <=.5 | .12 | ND | WT | I | S | I | S |
| *Candida albicans* | 2022 | <=1 | .25 | 32 | .12 | >4 | ND | NWT | S | R | S | R |
| *Candida albicans* | 2022 | <=1 | <=.12 | 1 | <=.06 | <=.12 | ND | NWT | S | S | S | S |
| *Candida tropicalis* | 2022 | <=1 | .25 | <=.5 | <=.06 | <=.12 | ND | NWT | S | S | S | S |
| *Candida albicans* | 2022 | 1 | .25 | .5 | 2 | .12 | ND | NWT | S | S | R | S |
| *Debaryomyces hansenii (Candida famata)* | 2022 |  |  |  |  |  | ND | ND | ND | ND | ND | ND |
| *Nakaseomyces glabratus (Candida glabrata)* | 2022 | <=1 |  |  | <=.06 | <=.12 | ND | NWT | ND | ND | S | S |
| *Nakaseomyces glabratus (Candida glabrata)* | 2022 | <=1 |  |  | <=.06 | <=.12 | ND | NWT | ND | ND | S | S |
| *Candida parapsilosis* | 2022 | <=1 | .25 | <=.5 | .5 | <=.12 | ND | NWT | S | S | S | S |
| *Nakaseomyces glabratus (Candida glabrata)* | 2022 | <=1 |  |  | <=.06 | <=.12 | ND | NWT | ND | ND | S | S |
| *Candida albicans* | 2022 | <=1 | <=.12 | <=.5 | <=.06 | <=.12 | ND | NWT | S | S | S | S |
| *Candida parapsilosis* | 2022 | <=1 | .5 | 1 | 1 | <=.12 | ND | NWT | S | S | S | S |
| *Candida albicans* | 2022 | <=1 | <=.12 | <=.5 | <=.06 | <=.12 | ND | NWT | S | S | S | S |
| *Candida albicans* | 2022 | <=1 | <=.12 | <=.5 | <=.06 | <=.12 | ND | NWT | S | S | S | S |
| *Candida tropicalis* | 2022 | <=1 | <=.12 | <=.5 | <=.06 | <=.12 | ND | NWT | S | S | S | S |
| *Candida albicans* | 2022 | <=1 | <=.12 | <=.5 | <=.06 | <=.12 | ND | NWT | S | S | S | S |
| *Candida albicans* | 2022 | <=1 | <=.12 | <=.5 | <=.06 | <=.12 | ND | NWT | S | S | S | S |
| *Candida parapsilosis* | 2022 | <=1 | .5 | 16 | 1 | .25 | ND | NWT | S | R | S | I |
| *Candida albicans* | 2022 | 2 | <=.12 | <=.5 | <=.06 | <=.12 | ND | NWT | S | S | S | S |
| *Nakaseomyces glabratus (Candida glabrata)* | 2022 | <=1 |  |  | <=.06 | <=.12 | ND | NWT | ND | ND | S | S |
| *Nakaseomyces glabratus (Candida glabrata)* | 2022 | <=1 |  |  | <=.06 | .25 | ND | NWT | ND | ND | S | I |
| *Candida parapsilosis* | 2022 | <=1 | .5 | <=.5 | .5 | <=.12 | ND | NWT | S | S | S | S |
| *Candida albicans* | 2022 | <=1 | <=.12 | <=.5 | <=.06 | <=.12 | ND | NWT | S | S | S | S |
| *Candida tropicalis* | 2022 | <=1 | <=.12 | <=.5 | <=.06 | <=.12 | ND | NWT | S | S | S | S |
| *Candida tropicalis* | 2022 | <=1 | <=.12 | <=.5 | <=.06 | <=.12 | ND | NWT | S | S | S | S |
| *Candida albicans* | 2022 | <=1 | <=.12 | <=.5 | <=.06 | <=.12 | ND | NWT | S | S | S | S |
| *Candida albicans* | 2022 | <=1 | <=.12 | 4 | <=.06 | <=.12 | ND | NWT | S | SDD | S | S |
| *Candida tropicalis* | 2022 | <=1 | <=.12 | <=.5 | <=.06 | <=.12 | ND | NWT | S | S | S | S |
| *Candida parapsilosis* | 2022 | <=1 | .5 | <=.5 | 1 | <=.12 | ND | NWT | S | S | S | S |
| *Candida parapsilosis* | 2022 | <=1 | >4 | 8 | .5 | <=.12 | ND | NWT | I | R | S | S |
| *Candida parapsilosis* | 2022 | <=1 | .25 | <=.5 | 1 | <=.12 | ND | NWT | S | S | S | S |
| *Candida albicans* | 2023 | <=1 | <=.12 | <=.5 | <=.06 | <=.12 | ND | NWT | S | S | S | S |
| *Candida albicans* | 2023 | <=1 | <=.12 | <=.5 | <=.06 | <=.12 | ND | NWT | S | S | S | S |
| *Candida albicans* | 2023 | <=1 | <=.12 | <=.5 | <=.06 | <=.12 | ND | NWT | S | S | S | S |
| *Candida albicans* | 2023 | <=1 | .25 | 16 | <=.06 | <=.12 | ND | NWT | S | R | S | S |
| *Candida albicans* | 2023 | <=1 | <=.12 | <=.5 | <=.06 | <=.12 | ND | NWT | S | S | S | S |
| *Candida albicans* | 2023 | <=1 | .25 | 8 | <=.06 | <=.12 | ND | NWT | S | R | S | S |
| *Candida albicans* | 2023 | <=1 | <=.12 | <=.5 | <=.06 | <=.12 | ND | NWT | S | S | S | S |
| *Candida parapsilosis* | 2023 | <=1 | .25 | <=.5 | .5 | <=.12 | ND | NWT | S | S | S | S |
| *Candida albicans* | 2023 | <=1 | <=.12 | <=.5 | <=.06 | <=.12 | ND | NWT | S | S | S | S |
| *Candida parapsilosis* | 2023 | <=1 | .25 | <=.5 | 1 | <=.12 | ND | NWT | S | S | S | S |
| *Candida parapsilosis* | 2023 | <=1 | .25 | <=.5 | 1 | <=.12 | ND | NWT | S | S | S | S |
| *Candida parapsilosis* | 2023 | <=1 | 1 | <=.5 | 1 | <=.12 | ND | NWT | S | S | S | S |
| *Candida albicans* | 2023 | <=1 | .25 | 1 | <=.06 | 1 | ND | NWT | S | S | S | R |
| *Candida tropicalis* | 2023 | <=1 | <=.12 | 32 | <=.06 | 1 | ND | NWT | S | R | S | R |
| *Candida albicans* | 2023 | <=1 | <=.12 | <=.5 | <=.06 | <=.12 | ND | NWT | S | S | S | S |
| *Candida albicans* | 2023 | <=1 | <=.12 | <=.5 | <=.06 | <=.12 | ND | NWT | S | S | S | S |
| *Candida tropicalis* | 2023 | <=1 | <=.12 | <=.5 | <=.06 | <=.12 | ND | NWT | S | S | S | S |
| *Candida albicans* | 2023 | <=1 | .5 | 8 | .5 | <=.12 | ND | NWT | I | R | I | S |
| *Candida tropicalis* | 2023 | <=1 | <=.12 | 1 | <=.06 | <=.12 | ND | NWT | S | S | S | S |
| *Candida tropicalis* | 2023 | <=1 | <=.12 | 1 | <=.06 | <=.12 | ND | NWT | S | S | S | S |
| *Candida tropicalis* | 2023 | <=1 | <=.12 | 1 | <=.06 | <=.12 | ND | NWT | S | S | S | S |
| *Candida albicans* | 2023 | <=1 | <=.12 | <=.5 | <=.06 | <=.12 | ND | NWT | S | S | S | S |
| *Candida albicans* | 2023 | <=1 | .25 | 32 | 4 | 4 | ND | NWT | S | R | R | R |
| *Candida albicans* | 2023 | <=1 | <=.12 | <=.5 | <=.06 | <=.12 | ND | NWT | S | S | S | S |
| *Candida parapsilosis* | 2023 | <=1 | .5 | <=.5 | 1 | <=.12 | ND | NWT | S | S | S | S |
| *Candida albicans* | 2023 | <=1 | <=.12 | <=.5 | <=.06 | <=.12 | ND | NWT | S | S | S | S |
| *Candida albicans* | 2023 | <=1 | <=.12 | <=.5 | <=.06 | <=.12 | ND | NWT | S | S | S | S |
| *Candida albicans* | 2023 | <=1 | <=.12 | <=.5 | <=.06 | <=.12 | ND | NWT | S | S | S | S |
| *Candida dubliniensis* | 2023 | <=1 | .25 | 16 |  | <=.12 | ND | NWT | S | R | ND | S |
| *Candida albicans* | 2023 | <=1 | <=.12 | 8 | <=.06 | <=.12 | ND | NWT | S | R | S | S |
| *Nakaseomyces glabratus (Candida glabrata)* | 2023 | <=1 |  |  | <=.06 | <=.12 | ND | NWT | ND | ND | S | S |
| *Candida albicans* | 2023 | >32 | <=.12 | 1 | <=.06 | <=.12 | ND | NWT | S | S | S | S |
| *Candida tropicalis* | 2023 | <=1 | <=.12 | 1 | <=.06 | <=.12 | ND | NWT | S | S | S | S |
| *Nakaseomyces glabratus (Candida glabrata)* | 2023 | <=1 |  |  | <=.06 | <=.12 | ND | NWT | ND | ND | S | S |
| *Candida parapsilosis* | 2023 | <=1 | <=.12 | <=.5 | <=.06 | <=.12 | ND | NWT | S | S | S | S |
| *Candida albicans* | 2023 | <=1 | <=.12 | <=.5 | <=.06 | <=.12 | ND | NWT | S | S | S | S |
| *Candida tropicalis* | 2023 | <=1 | <=.12 | <=.5 | <=.06 | <=.12 | ND | NWT | S | S | S | S |
| *Candida albicans* | 2023 | <=1 | <=.12 | <=.5 | <=.06 | <=.12 | ND | NWT | S | S | S | S |
| *Candida albicans* | 2023 | <=1 | <=.12 | <=.5 | <=.06 | <=.12 | ND | NWT | S | S | S | S |
| *Candida albicans* | 2023 | <=1 | >4 | 4 | 4 | <=.12 | ND | NWT | R | SDD | R | S |
| *Candida albicans* | 2023 | <=1 | <=.12 | <=.5 | <=.06 | <=.12 | ND | NWT | S | S | S | S |
| *Candida albicans* | 2023 | <=1 | <=.12 | <=.5 | <=.06 | <=.12 | ND | NWT | S | S | S | S |
| *Candida albicans* | 2023 | <=1 | <=.12 | 1 | <=.06 | <=.12 | ND | NWT | S | S | S | S |
| *Candida albicans* | 2023 | <=1 | <=.12 | 1 | <=.06 | <=.12 | ND | NWT | S | S | S | S |
| *Candida albicans* | 2023 | <=1 | <=.12 | 1 | <=.06 | <=.12 | ND | NWT | S | S | S | S |
| *Candida tropicalis* | 2023 | <=1 | <=.12 | 1 | <=.06 | <=.12 | ND | NWT | S | S | S | S |
| *Candida tropicalis* | 2023 | <=1 | <=.12 | <=.5 | <=.06 | <=.12 | ND | NWT | S | S | S | S |
| *Candida tropicalis* | 2023 | <=1 | .25 | 2 | <=.25 | <=.12 | ND | NWT | S | S | S | S |
| *Candida tropicalis* | 2023 | <=1 | <=.12 | 1 | <=.06 | <=.12 | ND | NWT | S | S | S | S |
| *Candida albicans* | 2023 | <=1 | <=.12 | <=.5 | <=.06 | <=.12 | ND | NWT | S | S | S | S |
| *Wickerhamomyces anomalus (Candida pelliculosa)* | 2023 | <=1 | .25 | 2 |  | <=.12 | ND | NWT | S | S | ND | S |
| *Candida albicans* | 2023 | <=1 | <=.12 | 1 | <=.06 | <=.12 | ND | NWT | S | S | S | S |
| *Candida albicans* | 2023 | <=1 | <=.12 | 1 | <=.06 | <=.12 | ND | NWT | S | S | S | S |
| *Candida albicans* | 2023 | <=1 | <=.12 | <=.5 | <=.06 | <=.12 | ND | NWT | S | S | S | S |
| *Candida albicans* | 2023 | <=1 | <=.12 | <=.5 | <=.06 | <=.12 | ND | NWT | S | S | S | S |
| *Candida albicans* | 2023 | <=1 | <=.12 | <=.5 | <=.06 | <=.12 | ND | NWT | S | S | S | S |
| *Candida parapsilosis* | 2023 | <=1 | .5 | <=.5 | .5 | <=.12 | ND | NWT | S | S | S | S |
| *Candida tropicalis* | 2023 | <=1 | <=.12 | <=.5 | <=.06 | <=.12 | ND | NWT | S | S | S | S |
| *Candida albicans* | 2023 | <=1 | <=.12 | <=.5 | <=.06 | <=.12 | ND | NWT | S | S | S | S |
| *Candida parapsilosis* | 2023 | <=1 | .25 | 1 | <=.06 | 1 | ND | NWT | S | S | S | R |
| *Candida auris* | 2023 |  |  |  |  |  | ND | ND | ND | ND | ND | ND |
| *Candida albicans* | 2023 | <=1 | <=.12 | 1 | <=.06 | <=.12 | ND | NWT | S | S | S | S |
| *Candida haemulonii* | 2023 | <=1 | .5 | 8 |  | <=.12 | ND | NWT | I | R | ND | S |
| *Candida parapsilosis* | 2023 | <=1 | .5 | 32 | .5 | .5 | ND | NWT | S | R | S | I |
| *Candida albicans* | 2023 | <=1 | <=.12 | <=.5 | <=.06 | <=.12 | ND | NWT | S | S | S | S |
| *Nakaseomyces glabratus (Candida glabrata)* | 2023 | <=1 |  |  | <=.06 | .5 | ND | NWT | ND | ND | S | I |
| *Candida albicans* | 2023 | <=1 | .25 | <=.5 | <=.06 | <=.12 | ND | NWT | S | S | S | S |
| *Candida albicans* | 2023 | 2 | <=.12 | 2 | <=.06 | <=.12 | ND | NWT | S | S | S | S |
| *Candida albicans* | 2024 | <=1 | .25 | 2 | <=.06 | <=.12 | ND | NWT | S | S | S | S |
| *Candida albicans* | 2024 | <=1 | <=.12 | <=.5 | <=.06 | <=.12 | ND | NWT | S | S | S | S |
| *Wickerhamomyces anomalus (Candida pelliculosa)* | 2024 | <=1 | 2 | 2 |  | .25 | ND | NWT | R | S | ND | I |
| *Candida parapsilosis* | 2024 | <=1 | .5 | 1 | .5 | <=.12 | ND | NWT | S | S | S | S |
| *Candida parapsilosis* | 2024 | <=1 | .25 | <=.5 | .5 | <=.12 | ND | NWT | S | S | S | S |
| *Candida albicans* | 2024 | <=1 | <=.12 | <=.5 | <=.06 | <=.12 | ND | NWT | S | S | S | S |
| *Nakaseomyces glabratus (Candida glabrata)* | 2024 | <=1 |  |  | <=.06 | <=.12 | ND | NWT | ND | ND | S | S |
| *Candida albicans* | 2024 | <=1 | <=.12 | <=.5 | <=.06 | <=.12 | ND | NWT | S | S | S | S |
| *Candida albicans* | 2024 | <=1 | <=.12 | <=.5 | <=.06 | <=.12 | ND | NWT | S | S | S | S |
| *Candida albicans* | 2024 | <=1 | <=.12 | <=.5 | <=.06 | <=.12 | ND | NWT | S | S | S | S |
| *Candida albicans* | 2024 | <=1 | <=.12 | <=.5 | <=.06 | <=.12 | ND | NWT | S | S | S | S |
| *Candida albicans* | 2024 | <=1 | <=.12 | <=.5 | <=.06 | <=.12 | ND | NWT | S | S | S | S |
| *Candida tropicalis* | 2024 | <=1 | <=.12 | 1 | <=.06 | <=.12 | ND | NWT | S | S | S | S |
| *Candida albicans* | 2024 | <=1 | .25 | 1 | <=.06 | <=.12 | ND | NWT | S | S | S | S |
| *Candida albicans* | 2024 | <=1 | <=.12 | <=.5 | <=.06 | <=.12 | ND | NWT | S | S | S | S |
| *Candida albicans* | 2024 | <=1 | <=.12 | <=.5 | <=.06 | <=.12 | ND | NWT | S | S | S | S |
| *Candida albicans* | 2024 | <=1 | <=.12 | <=.5 | <=.06 | <=.12 | ND | NWT | S | S | S | S |
| *Candida albicans* | 2024 |  |  |  |  |  | ND | ND | ND | ND | ND | ND |
| *Candida albicans* | 2024 | <=1 | <=.12 | 1 | <=.06 | <=.12 | ND | NWT | S | S | S | S |
| *Candida parapsilosis* | 2024 | <=1 | .5 | <=.5 | 1 | <=.12 | ND | NWT | S | S | S | S |
| *Candida tropicalis* | 2024 | <=1 | <=.12 | 1 | <=.06 | <=.12 | ND | NWT | S | S | S | S |
| *Candida albicans* | 2024 | <=1 | .25 | <=.5 | <=.06 | <=.12 | ND | NWT | S | S | S | S |
| *Candida albicans* | 2024 | <=1 | .25 | <=.5 | <=.06 | <=.12 | ND | NWT | S | S | S | S |
| *Candida parapsilosis* | 2024 | <=1 | .25 | 2 | 1 | <=.12 | ND | NWT | S | S | S | S |
| *Candida albicans* | 2024 | <=1 | .25 | <=.5 | <=.06 | <=.12 | ND | NWT | S | S | S | S |
| *Candida tropicalis* | 2024 | <=1 | <=.12 | <=.5 | <=.06 | <=.12 | ND | NWT | S | S | S | S |
| *Candida albicans* | 2024 | <=1 | .25 | <=.5 | <=.06 | <=.12 | ND | NWT | S | S | S | S |
| *Candida dubliniensis* | 2024 | <=1 | <=.12 | <=.5 |  | <=.12 | ND | NWT | S | S | ND | S |
| *Candida albicans* | 2024 | <=1 | <=.12 | <=.5 | <=.06 | <=.12 | ND | NWT | S | S | S | S |
| *Candida albicans* | 2024 | 2 | <=.12 | <=.5 | <=.06 | <=.12 | ND | NWT | S | S | S | S |
| *Candida albicans* | 2024 | <=1 | <=.12 | <=.5 | <=.06 | <=.12 | ND | NWT | S | S | S | S |
| *Nakaseomyces glabratus (Candida glabrata)* | 2024 | <=1 |  |  | <=.06 | .5 | ND | NWT | ND | ND | S | I |
| *Candida albicans* | 2024 | <=1 | .25 | <=.5 | <=.06 | <=.12 | ND | NWT | S | S | S | S |
| *Candida albicans* | 2024 | <=1 | .25 | <=.5 | <=.06 | <=.12 | ND | NWT | S | S | S | S |
| *Wickerhamomyces anomalus (Candida pelliculosa)* | 2024 | <=1 | <=.12 | 2 |  | .5 | ND | NWT | S | S | ND | I |
| *Nakaseomyces glabratus (Candida glabrata)* | 2024 | <=1 |  |  | <=.06 | <=.12 | ND | NWT | ND | ND | S | S |
| *Candida albicans* | 2024 | <=1 | <=.12 | 1 | <=.06 | <=.12 | ND | NWT | S | S | S | S |
| *Nakaseomyces glabratus (Candida glabrata)* | 2024 | <=1 |  |  | <=.06 | <=.12 | ND | NWT | ND | ND | S | S |
| *Nakaseomyces glabratus (Candida glabrata)* | 2024 | <=1 |  |  | <=.06 | <=.12 | ND | NWT | ND | ND | S | S |
| *Candida dubliniensis* | 2024 | <=1 | .25 | 8 |  | <=.12 | ND | NWT | S | R | ND | S |
| *Candida tropicalis* | 2024 | <=1 | <=.12 | 1 | <=.06 | <=.12 | ND | NWT | S | S | S | S |
| *Nakaseomyces glabratus (Candida glabrata)* | 2024 | <=1 |  |  | <=.06 | .25 | ND | NWT | ND | ND | S | I |
| *Candida albicans* | 2024 | <=1 | <=.12 | 1 | <=.06 | <=.12 | ND | NWT | S | S | S | S |
| *Nakaseomyces glabratus (Candida glabrata)* | 2024 | <=1 |  |  | <=.06 | .25 | ND | NWT | ND | ND | S | I |
| *Candida albicans* | 2024 | <=1 | <=.12 | <=.5 | <=.06 | <=.12 | ND | NWT | S | S | S | S |
| *Candida albicans* | 2024 | <=1 | <=.12 | <=.5 | <=.06 | <=.12 | ND | NWT | S | S | S | S |
| *Candida albicans* | 2024 | <=1 | <=.12 | 2 | <=.06 | <=.12 | ND | NWT | S | S | S | S |
| *Candida tropicalis* | 2024 | <=1 | <=.12 | 1 | <=.06 | <=.12 | ND | NWT | S | S | S | S |
| *Nakaseomyces glabratus (Candida glabrata)* | 2024 | <=1 |  |  | <=.06 | <=.12 | ND | NWT | ND | ND | S | S |
| *Metschnikowia pulcherrima (Candida pulcherrima)* | 2024 |  |  |  |  |  | ND | ND | ND | ND | ND | ND |
| *Candida albicans* | 2024 | <=1 | .5 | <=.5 | <=.5 | <=.12 | ND | NWT | I | S | I | S |
| *Candida albicans* | 2024 | <=1 | <=.12 | <=.5 | <=.06 | <=.12 | ND | NWT | S | S | S | S |
| *Candida parapsilosis* | 2024 | <=1 | .5 | <=.5 | 1 | <=.12 | ND | NWT | S | S | S | S |
| *Nakaseomyces glabratus (Candida glabrata)* | 2024 | <=1 |  |  | <=.06 | <=.12 | ND | NWT | ND | ND | S | S |
| *Candida tropicalis* | 2024 | <=1 | <=.12 | 1 | <=.06 | <=.12 | ND | NWT | S | S | S | S |
| *Candida tropicalis* | 2024 | <=1 | .25 | 1 | <=.06 | <=.12 | ND | NWT | S | S | S | S |
| *Candida tropicalis* | 2024 | <=1 | <=.12 | 1 | <=.06 | <=.12 | ND | NWT | S | S | S | S |
| *Candida albicans* | 2024 | <=1 | <=.12 | <=.5 | <=.06 | <=.12 | ND | NWT | S | S | S | S |
| *Candida albicans* | 2024 | <=1 | <=.12 | <=.5 | <=.06 | <=.12 | ND | NWT | S | S | S | S |
| *Candida tropicalis* | 2024 | <=1 | .25 | 1 | <=.06 | <=.12 | ND | NWT | S | S | S | S |
| *Candida albicans* | 2024 | <=1 | <=.12 | <=.5 | <=.06 | <=.12 | ND | NWT | S | S | S | S |
| *Candida albicans* | 2024 | <=1 | <=.12 | <=.5 | <=.06 | <=.12 | ND | NWT | S | S | S | S |
| *Candida albicans* | 2024 | <=1 | <=.12 | <=.5 | <=.06 | <=.12 | ND | NWT | S | S | S | S |
| *Candida albicans* | 2024 | <=1 | <=.12 | 1 | <=.06 | <=.12 | ND | NWT | S | S | S | S |
| *Candida parapsilosis* | 2024 | <=1 | 1 | <=.5 | 2 | <=.12 | ND | NWT | S | S | S | S |
| *Candida albicans* | 2024 | <=1 | <=.12 | <=.5 | <=.06 | <=.12 | ND | NWT | S | S | S | S |
| *Candida albicans* | 2024 | <=1 | <=.12 | 1 | <=.06 | <=.12 | ND | NWT | S | S | S | S |
| *Candida albicans* | 2024 | <=1 | <=.12 | <=.5 | <=.06 | <=.12 | ND | NWT | S | S | S | S |
| *Candida albicans* | 2024 | <=1 | <=.12 | 1 | <=.06 | <=.12 | ND | NWT | S | S | S | S |
| *Candida albicans* | 2024 | <=1 | <=.12 | <=.5 | <=.06 | <=.12 | ND | NWT | S | S | S | S |
| *Candida albicans* | 2024 | <=1 | <=.12 | <=.5 | <=.06 | <=.12 | ND | NWT | S | S | S | S |
| *Candida albicans* | 2024 | <=1 | <=.12 | 1 | <=.06 | 1 | ND | NWT | S | S | S | R |
| *Candida albicans* | 2024 | <=1 | <=.12 | <=.5 | <=.06 | <=.12 | ND | NWT | S | S | S | S |
| *Candida tropicalis* | 2024 | <=1 | <=.12 | 2 | <=.06 | <=.12 | ND | NWT | S | S | S | S |
| *Candida tropicalis* | 2024 | <=1 | <=.12 | <=.5 | <=.06 | <=.12 | ND | NWT | S | S | S | S |
| *Candida albicans* | 2024 | <=1 | <=.12 | <=.5 | <=.06 | <=.12 | ND | NWT | S | S | S | S |
| *Candida parapsilosis* | 2024 | <=1 | <=.12 | 1 | <=.06 | <=.12 | ND | NWT | S | S | S | S |
| *Candida albicans* | 2024 | <=1 | <=.12 | 4 | <=.06 | <=.12 | ND | NWT | S | SDD | S | S |
| *Candida albicans* | 2024 | <=1 | <=.12 | 16 | <=.06 | <=.12 | ND | NWT | S | R | S | S |
| *Candida albicans* | 2024 | <=1 | <=.12 | <=.5 | <=.06 | <=.12 | ND | NWT | S | S | S | S |
| *Candida albicans* | 2024 | <=1 | <=.12 | <=.5 | <=.06 | <=.12 | ND | NWT | S | S | S | S |
| *Candida parapsilosis* | 2024 | <=1 | <=.12 | 1 | <=.06 | <=.12 | ND | NWT | S | S | S | S |
| *Candida parapsilosis* | 2024 | <=1 | <=.12 | 1 | <=.06 | <=.12 | ND | NWT | S | S | S | S |
| *Candida albicans* | 2024 | <=1 | <=.12 | <=.5 | <=.06 | <=.12 | ND | NWT | S | S | S | S |
| *Candida albicans* | 2024 | <=1 | <=.12 | <=.5 | .12 | <=.12 | ND | NWT | S | S | S | S |
| *Candida tropicalis* | 2024 | <=1 | <=.12 | <=.5 | .12 | <=.12 | ND | NWT | S | S | S | S |
| *Nakaseomyces glabratus (Candida glabrata)* | 2024 | <=1 |  |  | <=.06 | <=.12 | ND | NWT | ND | ND | S | S |
| *Candida tropicalis* | 2024 | <=1 | <=.12 | <=.5 | <=.06 | <=.12 | ND | NWT | S | S | S | S |
| *Candida parapsilosis* | 2024 | 4 | .25 | 4 | .5 | <=.12 | ND | NWT | S | SDD | S | S |
| *Candida tropicalis* | 2024 | <=1 | <=.12 | 1 | <=.06 | <=.12 | ND | NWT | S | S | S | S |
| *Candida tropicalis* | 2024 | <=1 | <=.12 | 1 | <=.06 | <=.12 | ND | NWT | S | S | S | S |
| *Candida albicans* | 2024 | <=1 | <=.12 | <=.5 | <=.06 | <=.12 | ND | NWT | S | S | S | S |
| *Candida albicans* | 2024 | <=1 | <=.12 | <=.5 | <=.06 | <=.12 | ND | NWT | S | S | S | S |

Note: MIC values are expressed in µg/mL. ND, not determined/not reported; S, susceptible; SDD, susceptible dose-dependent; I, intermediate; R, resistant; WT, wild-type; NWT, non-wild-type. 5-fluorocytosine was interpreted using ECOFF-based WT/NWT categories only.
