# Supplementary material for: Clinical and microbiological epidemiology of Candida infections in a high-complexity hospital in Tolima, Colombia (2014–2024)
Source: PLoS One. 2026 Jul 24;21(7):e0354684. doi: 10.1371/journal.pone.0354684 (PMC13399354; doi:10.1371/journal.pone.0354684)
Supplement: S4 Table — (DOCX) [file pone.0354684.s008.docx]

**Supplementary. S4 Table.** Variables and coding used in multiple correspondence analysis (MCA).

**A) Clinical database MCA (episode-level records).**

| **Variable** | **Coding/categories** | **Missing/ND handling** | **Notes** |
| --- | --- | --- | --- |
| Type of Candida infection (clinical classification) | Candidemia; Unspecified candidiasis; Vulvovaginal candidiasis; Cutaneous/Nail candidiasis; Other organ-specific invasive records; CNS involvement/meningitis; Oropharyngeal mucocutaneous candidiasis; HIV-associated opportunistic candidiasis; Urinary candidiasis | ND/Unknown retained when present | Hospital database (episode-level) |
| Diagnosis name (ICD-10 label in database) | Candidemia; Skin and nail candidiasis; Candidiasis of other urogenital locations; Candidiasis of other sites; Unspecified candidiasis; Oropharyngeal candidiasis (Candidal stomatitis); Vulvovaginal candidiasis; HIV disease resulting in candidiasis; Fungal CNS involvement/meningitis due to *Candida* spp. | ND/Unknown retained when present | Hospital database (episode-level) |
| Sex | F; M | No missing category in MCA output | Hospital database |
| Age group | Early childhood; Childhood; Youth; Adolescence; Adulthood; Old age | No missing category in MCA output | Hospital database |
| Service/area of care | Emergency Department; Hospitalization; Surgery; Obstetrics; Adult ICU; Coronary Care Unit; Neonatal ICU | No missing category in MCA output | Hospital database |
| Sample/site category (as coded) | Blood; Bloodstream; Vulva, Vagina; Female genital tract; Oral cavity, tongue, palate, buccal mucosa, oropharynx, throat; Oropharyngeal cavity; Genitourinary; Urogenital; Superficial tissues/skin appendages; Skin, fingers, nails, legs, arms; Deep tissues/organs; Tissue, internal organs, bones, joints; Central nervous system; Cerebrospinal fluid (CSF); Multisite; Multiple organs/systems; Positive culture without site; N | ND retained as explicit category | Hospital database |
| Socioeconomic level | 1; 2; 3; 4; 5; 6; ND | No missing category in MCA output | Hospital database |
| Length of stay category (days) | 1-3; 4-10; 11-30; >30 | No missing category in MCA output | Hospital database |
| Binary clinical/procedural markers (0/1) | Variables: >27 YEARS OLD; ADULTHOOD; SHORT HOSPITAL STAY; INVASIVE DEVICES; SURGERY; TUMOR; RESPIRATORY DYSFUNCTION; CARDIOVASCULAR DISEASE. Coding: 0=absent, 1=present. | ND/Unknown retained when present | Hospital database |

**B) Laboratory database MCA (isolate-level records).**

| **Variable** | **Coding/categories** | **Missing/ND handling** | **Notes** |
| --- | --- | --- | --- |
| Clinical syndrome/type (as coded) | candidemia; Deep-seated non-candidemic invasive candidiasis; Vulvovaginal candidiasis; oropharyngeal mucocutaneous; Cutaneous/Nail; urinary; Respiratory; Digestive/intestinal; Unspecified candidiasis | ND/Unknown retained when present | Laboratory database (isolate-level) |
| Species (as reported) | *Candida albicans; Candida auris; Candida dubliniensis; Candida haemulonii; Candida parapsilosis; Candida tropicalis; Debaryomyces hansenii (Candida famata); Diutina catenulata (Candida catenulata); Metschnikowia pulcherrima (Candida pulcherrima); Meyerozyma guilliermondii (Candida guilliermondii); Nakaseomyces glabrata (Candida glabrata); Pichia kudriavzevii (Candida krusei); Wickerhamomyces anomalus (Candida pelliculosa); C. albicans; Non-albicans* | As recorded in laboratory database | Laboratory database |
| Specimen source (detailed; as coded) | Blood; Urine; Vulva, Vagina; Tracheal aspirate, bronchoalveolar lavage, sputum; Oral cavity, tongue, palate, buccal mucosa, oropharynx, throat; Skin, fingers, nails, legs, arms; Abdominal fluid, peritoneal fluid; Drainage, secretions, glands; Fecal matter; Ear canals, scalp; Positive culture without site | ND/Unknown retained when present | Laboratory database |
| Sample anatomical group (ANATOMICAL SAMPLE OF THE BODY) | Oropharyngeal cavity; Sterile serous cavities; Superficial cavities; ND; Surgical site/soft tissue; Superficial tissues/skin appendages; Bloodstream; Gastrointestinal tract; Female genitourinary tract; Lower respiratory tract; Urogenital | ND retained as explicit category | Laboratory database |
| Service/area (LOCATION NAME) | Surgery; Outpatient Clinic; Hospitalization; ND; Adult ICU; Coronary Care Unit; Neonatal ICU; Emergency Department | ND retained as explicit category | Laboratory database |
| Age group | Newborns; Early childhood; Childhood; Youth; Adolescence; Adulthood; Old age | No missing category in MCA output | As recorded in laboratory MCA dataset |
| Sex | F; M | No missing category in MCA output | As recorded in laboratory MCA dataset |
| Fluconazole interpretation | ND; R; S; SDD | ND = not tested/not reported | Laboratory database; WHONET-format exports (2022-2024) |
| Voriconazole interpretation | I; ND; R; S | ND = not tested/not reported | Laboratory database; WHONET-format exports (2022-2024) |
| Caspofungin interpretation | I; ND; R; S | ND = not tested/not reported | Laboratory database; WHONET-format exports (2022-2024) |
| Micafungin interpretation | I; ND; R; S | ND = not tested/not reported | Laboratory database; WHONET-format exports (2022-2024) |
| 5-flucytosine interpretation (WT/NWT) | ND; NWT; WT | ND = not tested/not reported | Laboratory database; WHONET-format exports (2022-2024) |
| Binary flags (0/1) | Variables: > 27 Adulthood; Male; Surgery; Candidemia; Invasive candidiasis; Urinary. Coding: 0=No, 1=Yes. | ND/Unknown retained when present | Laboratory MCA file |
