# Supplementary material for: Clinical and microbiological epidemiology of Candida infections in a high-complexity hospital in Tolima, Colombia (2014–2024)
Source: PLoS One. 2026 Jul 24;21(7):e0354684. doi: 10.1371/journal.pone.0354684 (PMC13399354; doi:10.1371/journal.pone.0354684)
Supplement: S5 Table — (DOCX) [file pone.0354684.s009.docx]

**Supplementary. S5 Table.** Top contributing categories to MCA dimensions.

**A)** Clinical database MCA: Dim1 = 9.1% inertia; Dim2 = 7.7% inertia.

| **Dimension** | **Variable/feature** | **Category level** | **Contribution (%)** | **Cos²** |
| --- | --- | --- | --- | --- |
| Dim1 (9.1%) | TYPE OF Candida INFECTION | Vulvovaginal candidiasis | 8.083 | 0.896 |
| Dim1 (9.1%) | DIAGNOSIS NAME | Vulvovaginal candidiasis | 8.083 | 0.896 |
| Dim1 (9.1%) | Sample/site | Female genital tract | 8.083 | 0.896 |
| Dim1 (9.1%) | Sample/site | Vulva, Vagina | 8.083 | 0.896 |
| Dim1 (9.1%) | Sex | M | 4.614 | 0.324 |
| Dim1 (9.1%) | TYPE OF Candida INFECTION | Mucocutaneous oropharyngeal | 4.612 | 0.333 |
| Dim1 (9.1%) | Sample/site | Oral cavity, tongue, palate, buccal mucosa, oropharynx, throat. | 4.612 | 0.333 |
| Dim1 (9.1%) | DIAGNOSIS NAME | Oropharyngeal candidiasis (Candidal stomatitis) | 4.612 | 0.333 |
| Dim1 (9.1%) | Sample/site | Oropharyngeal cavity | 4.612 | 0.333 |
| Dim1 (9.1%) | Age group | Youth | 3.627 | 0.262 |
| Dim2 (7.7%) | TYPE OF Candida INFECTION | Mucocutaneous oropharyngeal | 7.611 | 0.466 |
| Dim2 (7.7%) | Sample/site | Oropharyngeal cavity | 7.611 | 0.466 |
| Dim2 (7.7%) | Sample/site | Oral cavity, tongue, palate, buccal mucosa, oropharynx, throat. | 7.611 | 0.466 |
| Dim2 (7.7%) | Diagnosis name | Oropharyngeal candidiasis (Candidal stomatitis) | 7.611 | 0.466 |
| Dim2 (7.7%) | Sample/site | Bloodstream | 7.277 | 0.345 |
| Dim2 (7.7%) | Sample/site | Blood | 7.277 | 0.345 |
| Dim2 (7.7%) | Diagnosis name | Candidemia | 7.277 | 0.345 |
| Dim2 (7.7%) | Type of Candida infection *Candida* | Candidemia | 7.277 | 0.345 |
| Dim2 (7.7%) | Socioeconomic status | 1 | 4.311 | 0.229 |
| Dim2 (7.7%) | Age group | Early childhood | 3.419 | 0.177 |

**B)** Laboratory database MCA: Dim1 = 11.4% inertia; Dim2 = 7.3% inertia.

| **Dimension** | **Variable/feature** | **Category level** | **Contribution (%)** | **Cos²** |
| --- | --- | --- | --- | --- |
| Dim1 (11.4%) | 5-Fluorocytosine (FCT_NM) | ND | 6.812 | 0.93 |
| Dim1 (11.4%) | Voriconazole (VOR_NM) | ND | 6.812 | 0.93 |
| Dim1 (11.4%) | LOCATION NAME | ND | 6.452 | 0.826 |
| Dim1 (11.4%) | Micafungin (MIF_NM) | ND | 6.007 | 0.855 |
| Dim1 (11.4%) | Clinical syndrome | Cutaneous / Nail | 5.813 | 0.815 |
| Dim1 (11.4%) | ANATOMICAL SAMPLE OF THE BODY | Superficial tissues / skin appendages | 5.742 | 0.772 |
| Dim1 (11.4%) | Specimen source (detailed) | Skin, fingers, nails, legs, arms | 5.742 | 0.772 |
| Dim1 (11.4%) | Fluconazole (FLU_NM) | ND | 5.082 | 0.769 |
| Dim1 (11.4%) | Caspofungin (CAS_NM) | ND | 5.018 | 0.763 |
| Dim1 (11.4%) | 5-Fluorocytesine (FCT_NM) | NWT | 4.044 | 0.902 |
| Dim2 (7.2%) | Clinical syndrome | Candidemia | 10.728 | 0.624 |
| Dim2 (7.2%) | Candidemias | 1 | 10.728 | 0.624 |
| Dim2 (7.2%) | Specimen source (detailed) | Blood | 10.728 | 0.624 |
| Dim2 (7.2%) | ANATOMICAL SAMPLE OF THE BODY | Bloodstream | 10.728 | 0.624 |
| Dim2 (7.2%) | Invasive candidiasis | 1 | 8.495 | 0.586 |
| Dim2 (7.2%) | Specimen source (detailed) | Urine | 5.44 | 0.44 |
| Dim2 (7.2%) | ANATOMICAL SAMPLE OF THE BODY | Urogenital | 5.44 | 0.44 |
| Dim2 (7.2%) | Urinary | 1 | 5.44 | 0.44 |
| Dim2 (7.2%) | Clinical syndrome | Urinary | 5.44 | 0.44 |
| Dim2 (7.2%) | LOCATION NAME | Neonatal (ICU) | 3.286 | 0.179 |
