# Supplementary material for: Clinical and microbiological epidemiology of Candida infections in a high-complexity hospital in Tolima, Colombia (2014–2024)
Source: PLoS One. 2026 Jul 24;21(7):e0354684. doi: 10.1371/journal.pone.0354684 (PMC13399354; doi:10.1371/journal.pone.0354684)
Supplement: S6 Table — (DOCX) [file pone.0354684.s010.docx]

**Supplementary. S6 Table.** Descriptive 5-fluorocytosine profile according to ECOFF-based WT/NWT categories.

| ***Candida* spp.** | **Antifungal agent** | **WT n (%)** | **NWT n (%)** | **Total n (%)** |
| --- | --- | --- | --- | --- |
| *Candida albicans* | 5-Fluorocytosine | 1(0.93) | 106(99.06) | 107(100) |
| *Candida tropicalis* | 5-Fluorocytosine | - | 34(100) | 34(100) |
| *Candida parapsilosis* | 5-Fluorocytosine | - | 27(100) | 27(100) |
| *Nakaseomyces glabratus (C. glabrata)* | 5-Fluorocytosine | - | 19(100) | 19(100) |
| *Candidozyma haemuli (Candida haemulonii)* | 5-Fluorocytosine | - | 1(100) | 1(100) |
| *Candida dubliniensis* | 5-Fluorocytosine | - | 4(100) | 4(100) |
| *Meyerozyma guilliermondii (C. guilliermondii)* | 5-Fluorocytosine | 1(100) | - | 1(100) |
| *Wickerhamomyces anomalus (C. pelliculosa)* | 5-Fluorocytosine | - | 3(100) | 3(100) |

Note: 5-FC results are reported as ECOFF-based WT/NWT categories only; WT/NWT are not equivalent to clinical susceptible/resistant interpretations.
