# Supplementary material for: Clinical and microbiological epidemiology of Candida infections in a high-complexity hospital in Tolima, Colombia (2014–2024)
Source: PLoS One. 2026 Jul 24;21(7):e0354684. doi: 10.1371/journal.pone.0354684 (PMC13399354; doi:10.1371/journal.pone.0354684)
Supplement: Attachment — Submitted filename: Rebuttal Letter_R2.docx [file pone.0354684.s013.docx]

**Response to Reviewers**

PONE-D-26-11229R1 | PLOS ONE

Clinical and microbiological epidemiology of *Candida* infections in a high-complexity hospital in Tolima, Colombia (2014–2024)

Dear Academic Editor and Reviewers,

We would like to sincerely thank the Academic Editor and reviewers for the careful evaluation of our revised manuscript entitled **“Clinical and microbiological epidemiology of** Candida **infections in a high-complexity hospital in Tolima, Colombia (2014–2024)”.** We are grateful for the constructive comments and recommendations, which helped us further improve the clarity, consistency, and interpretation of the manuscript.

In this new revised version, we carefully addressed all additional comments raised during this review round. Specifically, we defined the abbreviations CLSI and EUCAST at first mention in the Abstract, checked and standardized the formatting of fungal species names throughout the manuscript, removed variables that were not sufficiently defined or further analyzed, clarified the interpretation of short hospital stay, expanded the Discussion of Candida species distribution, and reframed the interpretation of antifungal susceptibility findings according to clinical source. We also reviewed the reference list and the resubmission package according to PLOS ONE requirements.

We believe that the revised manuscript is clearer, more consistent, and more appropriately framed in relation to the available clinical and microbiological data. We again thank the Academic Editor and reviewers for their valuable feedback.

Sincerely,

Angel Gonzalez

On behalf of all authors

**1. PLOS ONE journal requirements and submission package**

| **N°** | **Source** | **Exact comment or request** | **Author's response** | **Change made / new page and line** |
| --- | --- | --- | --- | --- |
| JR-01 | PLOS submission package | Please include the following items when submitting your revised manuscript: A letter that responds to each point raised by the academic editor and reviewer(s), uploaded as a separate file labeled 'Response to Reviewers'; a marked-up copy of your manuscript that highlights changes made to the original version, uploaded as 'Revised Manuscript with Track Changes'; and an unmarked version of your revised paper without tracked changes, uploaded as 'Manuscript'. | *We thank PLOS ONE for these instructions. The revised submission package will include all required files: a point-by-point response letter addressing each comment raised by the Academic Editor and reviewers, a marked-up version of the revised manuscript showing the changes made, and a clean unmarked version of the manuscript.* | *The resubmission package will include the three required documents requested by PLOS ONE: “Response to Reviewers” (Rebuttal Letter_R2.docx), “Revised Manuscript with Track Changes” (Revised Marked Manuscript_R2.docx), and “Manuscript” (Clean Manuscript_R2.docx).*  *Location: resubmission package files.* |
| JR-02 | PLOS requirement | Please review your reference list to ensure that it is complete and correct. If you have cited papers that have been retracted, please include the rationale for doing so in the manuscript text, or remove these references and replace them with relevant current references. Any changes to the reference list should be mentioned in the rebuttal letter that accompanies your revised manuscript. If you need to cite a retracted article, indicate the article’s retracted status in the References list and also include a citation and full reference for the retraction notice. | *We thank PLOS ONE for this reminder. We reviewed the reference list to ensure completeness, consistency, and relevance. References added or retained during this revision were checked in relation to the revised discussion of species distribution and antifungal susceptibility. Any changes to the reference list are reflected in the revised manuscript and described in the corresponding responses*. | *The reference list was reviewed for completeness, consistency, and relevance. References supporting the expanded discussion of Candida species distribution and antifungal susceptibility were retained and cited where appropriate. Location: References section, pages 25–31; Discussion, pages 20–22.* |

**2. Additional Academic Editor comment**

| **N°** | **Source** | **Exact comment or request** | **Author's response** | **Change made / new page and line** |
| --- | --- | --- | --- | --- |
| ED-01 | Academic Editor | The reviewer has requested several minor revisions to improve the clarity and interpretation of the manuscript. Please carefully address the reviewer’s comments regarding terminology, variable definitions, risk factors, and the discussion of species distribution and antifungal susceptibility. | *We thank the Academic Editor for this guidance. We have carefully considered all reviewer comments and will address each requested revision to improve the clarity and interpretation of the manuscript. We will revise terminology, clarify variable definitions, refine the interpretation of risk factors, and strengthen the discussion of species distribution and antifungal susceptibility.* | *All reviewer comments will be addressed point by point in the revised manuscript and in the Response to Reviewers. Revisions will focus on terminology, variable definitions, risk factor interpretation, Candida species distribution, and antifungal susceptibility. Location: detailed changes are reported in the corresponding reviewer-specific responses below.* |

**3. Reviewer 1 comments**

| **N°** | **Source** | **Exact comment or request** | **Author's response** | **Change made / new page and line** |
| --- | --- | --- | --- | --- |
| R1-01 | Reviewer 1 | The authors have fully overhauled the interpretive framework of their paper. The revised manuscript is incredibly honest about its administrative and numeric boundaries, making it a highly accurate and useful baseline epidemiological survey for the Tolima region. No further revisions are required. | *We sincerely thank Reviewer 1 for this positive assessment of the revised manuscript. We are grateful that the reviewer recognizes the effort made to strengthen the interpretive framework, clearly acknowledge the administrative and numerical boundaries of the study, and present the work as an accurate and useful epidemiological baseline for the Tolima region* | *No additional manuscript changes were requested by Reviewer 1 for this comment* |

**4. Reviewer 2 comments**

| **N°** | **Source** | **Exact comment or request** | **Author's response** | **Change made / new page and line** |
| --- | --- | --- | --- | --- |
| R2-01 | Reviewer 2 | Abbreviations such as CLSI and EUCAST should be written in full at first mention in the abstract, as Clinical and Laboratory Standards Institute (CLSI) and European Committee on Antimicrobial Susceptibility Testing (EUCAST). | *We thank the reviewer for this observation. We revised the Abstract to write these abbreviations in full at first mention. Specifically, CLSI was defined as Clinical and Laboratory Standards Institute, and EUCAST was defined as European Committee on Antimicrobial Susceptibility Testing.* | *The Methods sentence in the Abstract was revised to define both abbreviations at first mention: “Clinical and Laboratory Standards Institute (CLSI)” and “European Committee on Antimicrobial Susceptibility Testing (EUCAST).” Location: Abstract, page 2, lines 24–26.* |
| R2-02 | Reviewer 2 | The formatting of fungal species names should be checked carefully and made consistent throughout the manuscript. In terms such as non-albicans *Candida*, the prefix “non-” should not be italicized, whereas albicans *Candida* should be italicized. | *We carefully checked the formatting of fungal species names throughout the manuscript. The prefix “non-” was kept in regular font, whereas the species epithet* albicans *and the genus name* Candida *were italicized where appropriate, including expressions such as non-*albicans Candida *and non-*albicans *isolation.* | *Fungal species names were checked and formatted consistently throughout the manuscript. The prefix “non-” was kept in regular font, whereas the species epithet* albicans *and the genus* Candida *were italicized where appropriate. Expressions such as non-*albicans *isolation, non-*albicans *species, and non-*albicans Candida *were revised accordingly. Location: throughout the manuscript, including Abstract, page 2, lines 30–39; Introduction, page 3, lines 50–57; Statistical analysis, page 7, lines 132 and 136; Results and Table 2, pages 14–16, lines 257–267; species distribution, pages 12–13, lines 204–225; and Conclusions, page 23-24, lines 420–434.* |
| R2-03 | Reviewer 2 | The variable socioeconomic strata appear in the results table, but its definition and interpretation are not clearly explained. The authors should clarify how this variable was categorized or scored, and whether a higher score indicates better or poorer socioeconomic status. If this variable is not further analyzed or discussed, it may be better to exclude it. | *We thank the reviewer for this observation. The socioeconomic strata variable was extracted as recorded in the hospital administrative database and was not generated or scored by the authors. No additional scoring system or analytical interpretation of this variable was available beyond the original hospital database categories. Because this variable was included only descriptively and was not further analyzed or discussed in the manuscript, we agree that it is clearer to exclude it to avoid ambiguity.* | *The socioeconomic strata variable was removed from Table 1, and the sentence describing its distribution was deleted from the Results section.*  *Location: Results section, page 9, lines 193–194; Table 1, page 10 and 11.* |
| R2-04 | Reviewer 2 | The section on predisposing factors should be removed or substantially revised, as these factors are not clearly included in the operational definitions and are not well explained in the discussion. For example, the term “predisposing medication” is too vague and should specify the type of medication. The discussion of short hospital stay is also unclear, particularly since most cases appear to involve mucocutaneous candidiasis, especially vulvovaginal candidiasis. | *We thank the reviewer for this important observation. We agree that the “predisposing factors” category, particularly the term “predisposing medications,” was too broad and was not sufficiently defined in the operational definitions. These variables were extracted as recorded in the hospital administrative database and were not available with enough granularity to specify medication classes or provide a robust analytical interpretation. Therefore, we removed the “Predisposing factors” section from the descriptive table. We also clarified the interpretation of short hospital stay. This variable is now interpreted cautiously and not as a protective factor; instead, the inverse association with clinically recorded candidemia likely reflects the predominance of mucocutaneous/non-invasive episodes, especially vulvovaginal candidiasis, among short-stay records.* | *The “Predisposing factors” section was removed from Table 1, including “predisposing medications,” “recent surgery,” “extended/prolonged hospital stay,” “short hospital stays,” “fever,” and “pregnancy or postpartum.” The Table 1 note was revised to remove reference to predisposing factors and now states only that comorbidities are not mutually exclusive. The Discussion was revised to clarify that the inverse association between short hospital stay and clinically recorded candidemia should not be interpreted as a protective effect, but rather as a case-mix signal related to the predominance of mucocutaneous/non-invasive episodes, particularly vulvovaginal candidiasis, in short-stay records.*  *Location: Table 1 note, page 11, lines 198–200; Discussion, pages 21–22, lines 384–388.* |
| R2-05 | Reviewer 2 | The discussion of *Candida* species distribution should be expanded. The authors should further explore the predominance of *Candida* albicans or non-albicans *Candida* species and compare these findings with previous studies. | *We thank the reviewer for this helpful suggestion. We expanded the Discussion of Candida species distribution to better interpret the predominance of C. albicans and the substantial proportion of non-albicans Candida species. We now explain that the predominance of C. albicans is consistent with its recognized role in mucocutaneous candidiasis and hospital-associated Candida infections, particularly because the clinical spectrum in this study was dominated by mucocutaneous disease, especially vulvovaginal and oropharyngeal candidiasis. We also discuss the non-albicans Candida fraction, mainly represented by C. parapsilosis, C. tropicalis, and N. glabratus, and compare this pattern with previous studies describing an epidemiological shift toward non-albicans Candida species in healthcare-associated settings.* | *The Discussion was expanded to further interpret and compare the observed Candida species distribution. The revised paragraph now addresses the predominance of C. albicans, the substantial proportion of non-albicans Candida species, the role of mucocutaneous disease in explaining the observed distribution, and the greater representation of non-albicans Candida species in critical care areas. Four previous studies were cited and discussed to support the comparison with reported epidemiological shifts toward non-albicans Candida species in healthcare-associated settings [26,30–32].*  *Location: Discussion, pages 20–21, lines 355–367.* |
| R2-06 | Reviewer 2 | The discussion of antifungal susceptibility requires clearer framing. Although many isolates appear to be susceptible to echinocandins, the discussion should not focus too heavily on invasive candidiasis if most isolates were not obtained from invasive cases. The authors should clearly distinguish the interpretation of antifungal susceptibility findings between invasive candidiasis and mucocutaneous candidiasis to avoid overstating the clinical implications. | *We thank the reviewer for this important comment. We agree that the antifungal susceptibility findings required clearer framing because most isolates were obtained from mucocutaneous, superficial, or urogenital sources rather than invasive specimens. We revised the Discussion to distinguish the interpretation of susceptibility results according to clinical source. The pooled susceptibility data are now framed primarily as descriptive microbiological surveillance data. For mucocutaneous and urogenital candidiasis, the findings are interpreted as species-level susceptibility patterns and possible azole variability. For invasive candidiasis, we clarify that the bloodstream subgroup was small and that echinocandin activity should be interpreted cautiously as an isolate-level signal consistent with international surveillance, not as independent evidence to guide empiric therapy or define institutional treatment protocols.* | *The antifungal susceptibility paragraph in the Discussion was substantially revised. The new text clearly distinguishes the interpretation of pooled susceptibility findings between mucocutaneous/urogenital candidiasis and invasive candidiasis. It emphasizes that most isolates came from mucocutaneous, superficial, or urogenital sources; therefore, pooled results should be interpreted primarily as descriptive microbiological surveillance data. The discussion of echinocandin activity was reframed cautiously to avoid overstating implications for invasive candidiasis, and the manuscript now states that these data should not be used alone to guide empiric therapy or define institutional treatment protocols.*  *Location: Discussion, antifungal susceptibility paragraph, pages 22, lines 389–406.* |
