# Supplementary material for: Clinical and microbiological epidemiology of Candida infections in a high-complexity hospital in Tolima, Colombia (2014–2024)
Source: PLoS One. 2026 Jul 24;21(7):e0354684. doi: 10.1371/journal.pone.0354684 (PMC13399354; doi:10.1371/journal.pone.0354684)
Supplement: Attachment — Submitted filename: Rebuttal Letter.docx [file pone.0354684.s012.docx]

**Response to Reviewers**

Working Matrix
PONE-D-26-11229 | PLOS ONE
Clinical and microbiological epidemiology of *Candida* infections in a high-complexity hospital in Tolima, Colombia (2014–2024)

Dear Academic Editor and Reviewers,

We would like to sincerely thank you for the careful evaluation of our manuscript entitled “Clinical and microbiological epidemiology of *Candida* infections in a high-complexity hospital in Tolima, Colombia (2014–2024)” (PONE-D-26-11229). We are grateful for the constructive comments and recommendations, which helped us improve the clarity, methodological rigor, clinical interpretation, and overall presentation of the study. We have carefully revised the manuscript in response to each comment raised by the Academic Editor and both reviewers. In particular, we clarified the relationship between the clinical/administrative and microbiological data sources, explicitly stating that they originated from the same participating hospital but were not linked record by record. We also revised the interpretation of the multivariable models, now framing them as exploratory and data-source specific rather than as definitive patient-level clinical–microbiological risk models.

The Methods section was substantially revised to improve transparency regarding study design, operational definitions, data management, antifungal susceptibility testing, VITEK 2 card use, WHONET MIC exports, and the interpretation of CLSI/EUCAST-based susceptibility categories. We also refined the classification of invasive candidiasis to distinguish candidemia and deep-seated non-candidemic invasive candidiasis, while explicitly acknowledging the limitations associated with other/unspecified clinical records. The Results, Discussion, and Conclusions were also revised to provide a more cautious and balanced interpretation of the findings. We tempered claims regarding clinical risk factors, empiric therapy, and institutional treatment protocols, and reframed the study as a longitudinal clinical–microbiological survey and local surveillance baseline.

In addition, we updated the tables, figures, figure legends, supplementary materials, Data Availability statement, repository information, licenses, and author metadata. The revised submission package includes a point-by-point Response to Reviewers, a marked version of the revised manuscript showing the changes made, and a clean version of the manuscript. De-identified datasets, R scripts, supporting workbooks, and reproducibility materials have been made available through Zenodo and GitHub. The authors’ ORCID identifiers have also been updated as follows:

Rafael Augusto Ramírez-Trujillo: 0000-0001-9355-1765

Paula Carvajal Hernández: 0000-0002-5024-1019

Ángel González: 0000-0002-7052-7938

We believe that the revised manuscript is clearer, more methodologically transparent, and more appropriately framed in relation to the available data. We again thank the Academic Editor and reviewers for their valuable feedback and hope that the revised version adequately addresses all concerns raised during the review process.

Sincerely,

On behalf of all authors

1. PLOS ONE journal requirements and submission package

| **N°** | **Source** | **Exact comment or request** | **Author's response** | **Change made / new page and line** |
| --- | --- | --- | --- | --- |
| JR-01 | PLOS requirement | Please ensure that your manuscript meets PLOS ONE's style requirements, including those for file naming. The PLOS ONE style templates can be found at  <https://journals.plos.org/plosone/s/file?id=wjVg/PLOSOne_formatting_sample_main_body.pdf>  <https://journals.plos.org/plosone/s/file?id=ba62/PLOSOne_formatting_sample_title_authors_affiliations.pdf> | *We thank PLOS ONE for this reminder. We reviewed the revised manuscript according to the PLOS ONE style templates for the main manuscript body and title/authors/affiliations. Formatting was checked and adjusted where necessary, including title page structure, author affiliations, headings, table and figure citations, table formatting, references, and file naming for the resubmission package.* | *Formatting was checked and adjusted throughout the manuscript and resubmission files according to PLOS ONE requirements. Location: throughout the manuscript and resubmission files.* |
| JR-02 | PLOS requirement | Please note that PLOS One has specific guidelines on code sharing for submissions in which author-generated code underpins the findings in the manuscript. In these cases, we expect all author-generated code to be made available without restrictions upon publication of the work. Please review our guidelines at  -<https://journals.plos.org/plosone/s/materials-and-software-sharing#loc-sharing-code>  and ensure that your code is shared in a way that follows best practice and facilitates reproducibility and reuse. | *We thank PLOS ONE for this reminder. The author-generated code, R scripts, and supporting workbooks used to generate the analyses, tables, and figures have been made publicly available without restrictions through Zenodo and GitHub to facilitate reproducibility and reuse.*  -https://doi.org/10.5281/zenodo.20817342  -<https://github.com/rafaelramirezt-del/candida-tolima-2014-2024.git> | *The Data Availability statement was updated to indicate that the analysis code, R scripts, and supporting workbooks are publicly available without restrictions at Zenodo and GitHub. Location: Data Availability section, page 24, lines 439–449* |
| JR-03 | PLOS requirement | We note that you have indicated that there are restrictions to data sharing for this study. For studies involving human research participant data or other sensitive data, we encourage authors to share de-identified or anonymized data. However, when data cannot be publicly shared for ethical reasons, we allow authors to make their data sets available upon request. For information on unacceptable data access restrictions, please see  <http://journals.plos.org/plosone/s/data-availability#loc-unacceptable-data-access-restrictions>. Before we proceed with your manuscript, please address the following prompts:  a) If there are ethical or legal restrictions on sharing a de-identified data set, please explain them in detail (e.g., data contain potentially identifying or sensitive patient information, data are owned by a third-party organization, etc.) and who has imposed them (e.g., a Research Ethics Committee or Institutional Review Board, etc.). Please also provide contact information for a data access committee, ethics committee, or other institutional body to which data requests may be sent.  b) If there are no restrictions, please upload the minimal anonymized data set necessary to replicate your study findings to a stable, public repository and provide us with the relevant URLs, DOIs, or accession numbers. Please see  <http://www.bmj.com/content/340/bmj.c181.long>  for guidelines on how to de-identify and prepare clinical data for publication. For a list of recommended repositories, please see <https://journals.plos.org/plosone/s/recommended-repositories>  . You also have the option of uploading the data as Supporting Information files, but we would recommend depositing data directly to a data repository if possible.  Please update your Data Availability statement in the submission form accordingly. | *We thank PLOS ONE for this clarification. We have deposited the minimal de-identified clinical and microbiological datasets required to reproduce the main analyses in Zenodo. These files are available together with the analysis code, R scripts, and supporting workbooks. The publicly available datasets do not include direct personal identifiers or sensitive institutional variables, such as patient names, identification numbers, addresses, bed numbers, room numbers, or floor information. The original individual-level institutional records remain under the custody of the participating institution and are not publicly shared due to ethical, legal, and institutional restrictions. The Data Availability statement was updated accordingly in the manuscript and will also be updated in the submission form.* | *The Data Availability statement was updated to indicate that de-identified clinical and microbiological datasets, together with the analysis code, R scripts, and supporting workbooks, are publicly available in Zenodo. The statement also clarifies that the public datasets do not include direct personal identifiers or sensitive institutional variables, and that the original institutional records remain restricted. Location: Data Availability statement, pages 24 and 25, lines 438-449.* |
| JR-04 | PLOS requirement | Your ethics statement should only appear in the Methods section of your manuscript. If your ethics statement is written in any section besides the Methods, please move it to the Methods section and delete it from any other section. Please ensure that your ethics statement is included in your manuscript, as the ethics statement entered into the online submission form will not be published alongside your manuscript. | *We thank PLOS ONE for this clarification. The Ethics statement was moved to the Methods section of the manuscript and deleted from its previous location outside the Methods section.* | *The Ethics statement was moved to the Methods section and revised to clarify that the original institutional records were accessed under restricted institutional conditions, whereas only de-identified datasets without direct personal identifiers or sensitive institutional variables were deposited for public sharing. Location: Methods section, page 7, lines 137–146.* |
| JR-05 | PLOS requirement | If the reviewer comments include a recommendation to cite specific previously published works, please review and evaluate these publications to determine whether they are relevant and should be cited. There is no requirement to cite these works unless the editor has indicated otherwise. | *We thank PLOS ONE for this clarification. We reviewed the references and guidelines suggested in the reviewers’ comments and incorporated those that were relevant to support the revised methodological definitions, antifungal susceptibility interpretation, and discussion of limitations.* | *Reviewer-suggested references and guidelines were reviewed, and relevant sources were incorporated where appropriate in the Methods, Discussion, and References sections. Location: Methods, Discussion, and References sections,* |
| JR-06 | PLOS submission package | Please include the following items when submitting your revised manuscript: A letter that responds to each point raised by the academic editor and reviewer(s). You should upload this letter as a separate file labeled 'Response to Reviewers'. A marked-up copy of your manuscript that highlights changes made to the original version. You should upload this as a separate file labeled 'Revised Manuscript with Track Changes'. An unmarked version of your revised paper without tracked changes. You should upload this as a separate file labeled 'Manuscript'. | *We thank PLOS ONE for these instructions. The revised submission package has been prepared with the required files: a point-by-point Response to Reviewers, a marked-up version of the revised manuscript showing the changes made, and a clean unmarked version of the manuscript.* | *The required resubmission files were prepared as separate documents: “Response to Reviewers,* *Rebuttal Letter” “Revised Manuscript with Track Changes,* *Revised Marked Manuscript” and “Manuscript,* *Clean Manuscript” Location: resubmission package files.* |

2. Additional Academic Editor comment

| **N°** | **Source** | **Exact comment or request** | **Author's response** | **Change made / new page and line** |
| --- | --- | --- | --- | --- |
| ED-01 | Academic Editor | The reviewers have evaluated this 10-year study on *Candida* infections in Tolima, Colombia. Key concerns include the need for clearer correlations between clinical and microbiological data, an explicit description of antifungal susceptibility testing methods, and clarification of discrepancies in candidemia incidence rates relative to national averages. Additionally, the study’s broad scope appears to limit the depth of analysis in specific areas. Overall, while the study provides valuable insights, it would benefit from greater methodological clarity and improved data analysis. | *We thank the Academic Editor for this summary and for recognizing the value of the study. We revised the manuscript to improve methodological clarity, particularly regarding the relationship between the clinical/administrative and microbiological data sources, antifungal susceptibility testing methods, candidemia count interpretation, and the overall scope of the conclusions. These changes are addressed in detail in the point-by-point responses below.* | *The manuscript was revised in the Methods, Results, Discussion, Conclusions, Data Availability statement, and relevant tables to improve methodological clarity and temper the clinical interpretation of the findings. The revisions clarified the relationship between the clinical/administrative and microbiological data sources, antifungal susceptibility testing methods, candidemia count interpretation, the exploratory nature of the multivariable models, and the overall scope of the conclusions. Location: Methods, pages 4–7, lines 73–136; Results, pages 8–18, lines 148–297; Discussion, pages 18–22, lines 299–402; Conclusions, page 23, lines 404–418; Data Availability, pages 24–25, lines 438–448; Tables 2–3, pages 15–18.* |

3. Reviewer 1 comments – matrix to complete

| **N°** | **Source** | **Exact comment or request** | **Author's response** | **Change made / new page and line** |
| --- | --- | --- | --- | --- |
| R1-GA | Reviewer 1 | This 10-year longitudinal study provides essential epidemiological data on *Candida* infections in Tolima, Colombia. The authors successfully integrated clinical and microbiological databases to characterize a sustained institutional burden. The statistical approach is robust, employing Firth-penalized regression to address the low frequency of candidemia events. The commitment to open science, demonstrated by the inclusion of Zenodo and GitHub repositories for code and data, is commendable. | *We thank Reviewer 1 for the positive assessment of our study and for recognizing the value of the 10-year epidemiological data, the use of clinical and microbiological sources, the statistical approach, and the open-science materials deposited in Zenodo and GitHub. We carefully revised the manuscript according to the reviewer’s major and minor comments, and the specific changes are detailed in the responses below.* | *No specific manuscript change was required for this general assessment. The revisions derived from Reviewer 1’s specific comments are detailed in the corresponding responses below.* |
| R1-01 | Reviewer 1 | 1. Database Linkage and Clinical Correlation The authors explicitly state that the clinical and microbiological databases were analyzed separately without individual-patient level linkage. While this is a common limitation in retrospective studies, it creates a gap in correlating clinical syndromes directly with specific fungal etiologies. Interestingly, the clinical database identified 20 candidemia cases, while the microbiological database identified 23 blood isolates. Recommendation: To improve the technical soundness of the "Associated Factors" section (Page 11, Lines 177–184), the authors should clarify if the 20 candidemia cases used for the multivariable model are subset from the same patient group as the 23 blood isolates. If linkage was performed for this specific subset, it should be stated. | *We thank the reviewer for this important observation. We clarified in the Methods that the clinical/administrative and microbiological data sources came from the same participating hospital but were generated through separate institutional information workflows and were not linked record by record. Therefore, the 20 clinical/administrative candidemia records and the 23 microbiological blood-isolate records should not be interpreted as directly matched events or as the same denominator. We also revised the Results section to make this distinction explicit.* | *The Methods section was revised to clarify that the clinical/administrative and microbiological databases came from the same participating hospital but were generated through separate institutional information workflows and were not linked record by record. The Methods section also clarifies the specific use of each data source: clinical/administrative data for clinically recorded candidiasis episodes and factors associated with candidemia and invasive candidiasis, and microbiological data for isolate-level analyses, species distribution, antifungal susceptibility, and non-albicans isolation. The Results section was revised to clarify that the 23 microbiological blood-isolate records were not linked record by record to the 20 clinical/administrative candidemia records and should be interpreted as complementary, non-matched counts. Location: Methods section, page 4, lines 73–81; page 5, lines 82–91; Results section, pages 12–13, lines 214–220.* |
| R1-02 | Reviewer 1 / | 2. 5-Fluorocytosine (5-FC) Susceptibility Anomaly Table 3 (Page 13) shows that 99.06% (106/107) of *C. albicans* isolates were classified as Non-Wild Type (NWT) for 5-FC. As the authors correctly note in the Discussion (Page 18, Lines 291–296), *C. albicans* is typically highly susceptible to 5-FC. Attributing this to a "surveillance artifact" related to system configuration or cutoff availability is a fair assessment. Recommendation: To ensure this doesn't mislead clinical readers, the authors should specify the VITEK 2 AST card version used and confirm if WHONET expert rules were applied. If the data is an artifact, consider moving the 5-FC results to a Supplementary Table to keep the focus of Table 3 on clinically relevant susceptibilities. | *We thank the reviewer for this important observation. We agree that the 5-fluorocytosine results, particularly the high proportion of non-wild-type classifications among C. albicans isolates, could be misleading if interpreted as clinical resistance. We revised the Methods section to specify the VITEK 2 cards used by the Microbiology Laboratory: yeast identification was performed using the YST card, and antifungal susceptibility testing was performed using the AST-YS08 card. We also clarified that MIC data obtained from VITEK 2 were exported and organized in WHONET format for data management, quality control, and analysis.*  *Susceptibility categories were interpreted according to updated CLSI criteria when clinical breakpoints were available, whereas EUCAST ECOFF-based criteria were used for descriptive WT/NWT interpretation when clinical breakpoints were not available. To avoid overinterpretation, 5-FC results were moved from the main susceptibility table to S6 Table and are now presented only as descriptive ECOFF-based WT/NWT categories, not as clinical S/I/R interpretations.* | *The Methods section was revised to specify that yeast identification was performed using the VITEK 2 YST card and antifungal susceptibility testing using the VITEK 2 AST-YS08 card during 2022–2024. The Methods section also clarifies that MIC data obtained from VITEK 2 were exported and organized in WHONET format for data management, quality control, and analysis, and that susceptibility categories were interpreted according to updated CLSI criteria when clinical breakpoints were available, whereas EUCAST ECOFF-based criteria were used for descriptive WT/NWT interpretation when clinical breakpoints were not available. The Results section and Table 3 were revised to keep the main susceptibility table focused on clinically interpretable S/SDD/I/R categories. Five-fluorocytosine results were moved from Table 3 to Supplementary Table 6 and are now reported only as descriptive ECOFF-based WT/NWT categories.* *Location: Methods section, page 6, lines 108–120; Results section, pages 15–16, lines 267–280; Table 3, pages 16–18, lines 281–290; S6 Table, page 31, line 600.* |
| R1-03 | Reviewer 1 | 3. Temporal Trend Interpretation Figure 1A (Page 28) and line 113 (Page 6) identify a peak in 2018 followed by a significant decline through 2024. In the Discussion (Page 16, Lines 241–242), the authors suggest this might reflect changes in coding or clinical criteria rather than true incidence. Recommendation: Given the administrative nature of the clinical database, the authors should explicitly state if there were changes to the Hospital Information System (HIS) or a shift from ICD-9 to ICD-10 coding during the 2018–2019 period that might have impacted case capture. | *We thank the reviewer for this important observation. We verified that during the 2018–2019 period the hospital did not change its information system/ software, and no shift from ICD-9 to ICD-10 coding occurred. We revised the Discussion to clarify that the 2018 peak and subsequent decline should not be attributed to a documented change in the Hospital Information System or coding system. However, because this was a record-based retrospective study, we emphasized that temporal trends should still be interpreted cautiously, as changes in clinical awareness, diagnostic capacity, sampling practices, coding intensity, infection control measures, or antimicrobial optimization initiatives may influence case capture without necessarily reflecting true changes in incidence.* | *The Discussion was revised to clarify that no hospital information software change or ICD coding transition occurred during 2018–2019, while emphasizing that temporal trends should be interpreted cautiously because administrative case capture may vary over time.* *Location: Discussion section, page 19, lines 315–324.* |
| R1-04 | Reviewer 1 | 4. Statistical Transparency in Regression Models For Table 2 (Page 12), please include the final N (number of cases included) in the footer for each model (Candidemia, Invasive Candidiasis, and Non-*albicans* isolation). This allows reviewers to verify the ratio of events to independent variables. | *We thank the reviewer for this helpful suggestion. We revised the footnote of Table 2 to include the final model-specific sample size and number of events for each multivariable model: candidemia, invasive candidiasis, and non-albicans isolation. This clarification allows readers to assess the number of observations and events included in each model according to its corresponding data source and outcome.* | *The footnote of Table 2 was revised to include the model-specific sample size and number of events for the candidemia, invasive candidiasis, and non-albicans isolation models. Location: Table 2 footnote, page 15, lines 262–266.* |
| R1-05 | Reviewer 1 | 5. Major Concern: Candidemia Incidence and Database Discrepancy The study reports 20 candidemia episodes identified in the clinical database and 23 blood isolates identified in the microbiological database over an 11-year period (2014–2024) across 243,013 hospitalizations. This results in an incidence rate of approximately 0.08 per 1,000 admissions, which is significantly lower than national Colombian averages (~1.96 per 1,000) and regional Latin American benchmarks (0.74 to 6.0 per 1,000 admissions). I have the following concerns and requests for clarification: • Internal Consistency: Why is there a discrepancy between the 20 clinical episodes and the 23 laboratory isolates? Does this imply that three confirmed laboratory cases were not captured in the administrative/clinical records? Please clarify if any manual validation was performed to ensure that the 20 cases used in the multivariable model represent the full clinical reality of the hospital. • Definition of "High-Complexity": The extremely low incidence (less than 2 cases per year) seems atypical for a high-complexity facility. To provide better context for these numbers, please include specific institutional data in the Methods section, such as the total number of ICU beds and the presence of specialized services (e.g., oncology, transplantation, or neonatal ICU) during the study period. • Information Bias: While the authors mention that fungal infections are underreported due to a lack of mandatory notification, the low count suggests a potential "information bias" stemming from the reliance on administrative ICD-10 coding. Please expand the Study Limitations to discuss how this specific reliance might have led to an underestimation of the invasive disease burden and how it may impact the power of the multivariable risk factor analysis. | *We thank the reviewer for this important comment. We agree that the low recorded candidemia rate requires careful clarification. We revised the Methods to clarify that the primary scope of the study was the overall clinical and microbiological burden of Candida infections, including mucocutaneous, localized, invasive, and bloodstream presentations, rather than candidemia alone. Therefore, candidemia represented a small bloodstream subset within the broader candidiasis dataset.*  *Regarding the apparent discrepancy between the 20 clinical/administrative candidemia records and the 23 microbiological blood-isolate records, we clarified that both data sources came from the same participating hospital but were generated through separate institutional information workflows and were not linked record by record for this retrospective study. Therefore, these counts should not be interpreted as matched events, and the difference does not necessarily imply that exactly three laboratory-confirmed cases were missed in the clinical/administrative database. No patient-level manual linkage between both sources was performed; consequently, the 20 clinical/administrative candidemia records should be interpreted as the retrospectively captured candidemia burden in the clinical/administrative database, not as the complete laboratory-confirmed candidemia burden of the hospital.*  *We also revised the Discussion to explicitly state that the low recorded candidemia rate may reflect undercapture of invasive fungal disease due to reliance on retrospective diagnostic coding and the lack of record-by-record linkage with laboratory-confirmed bloodstream isolates. Accordingly, the candidemia model is now interpreted as an exploratory analysis based on clinically recorded administrative data rather than as a definitive patient-level clinical–microbiological inference. Detailed bed-capacity indicators, such as total hospital beds or ICU beds, were not available as consistently verifiable variables within the analyzed databases across the full 2014–2024 period; therefore, we did not introduce non-analyzed institutional capacity data into the Results. Instead, we clarified the study scope, data-source structure, and limitations affecting interpretation of candidemia-specific estimates.* | *The Methods section was revised to clarify that the primary scope of the study was the overall clinical and microbiological burden of Candida infections rather than candidemia alone, and that the clinical/administrative and microbiological data sources were generated through separate institutional workflows and were not linked record by record. The Discussion was revised to clarify that the low recorded candidemia rate should be interpreted cautiously because candidemia capture depended on retrospective diagnostic coding and may underestimate the true invasive disease burden. The interpretation of the candidemia model was also softened, indicating that it should be considered an exploratory analysis rather than a definitive patient-level clinical–microbiological inference. Location: Methods section, page 4, lines 73–81; page 5, lines 82–91; Results section, page 8, lines 148–159; Results section, pages 12–13, lines 214–220; Discussion section, pages 18–19, lines 308–315; Discussion section, page 19, lines 315–323.* |
| R1-06 | Reviewer 1 | 6. Major Concern: Statistical Validity of Antifungal Susceptibility Testing (AFST) While the authors provide MIC data for 196 isolates, the sample sizes for several high-priority species are critically low, which introduces a high risk of selection bias and limits the validity of the reported resistance percentages. • Species-Level Fragility: In Table 3, susceptibility profiles for species like *Nakaseomyces glabratus* (n=19), *Candida dubliniensis* (n=4), and *Wickerhamomyces anomalus* (n=3) are based on numbers too small to represent institutional or regional trends. E.g. For *W. anomalus*, a single resistant isolate results in a reported resistance rate of 33.33%. • Bloodstream Etiology: The bloodstream subgroup (n=23) is even more fragmented, with only one *C. auris* isolate and two *N. glabratus* isolates identified. • Impact on Guidelines: The manuscript concludes that these findings support "susceptibility-guided therapy" and inform "local protocols". However, basing institutional protocols on such a limited number of isolates is statistically risky. Recommendation to Authors: Please include a clear disclaimer in the Results and Discussion stating that the susceptibility percentages for species with n < 30 (consistent with CLSI M101 guidelines for cumulative antibiograms) should be interpreted as individual case signals rather than representative institutional resistance rates. The authors should also clarify if these isolates were collected from the same patient (duplicate isolates) or represent unique infection episodes. | *We thank the reviewer for this important observation. We agree that susceptibility percentages based on small species-specific denominators should not be interpreted as representative institutional or regional resistance rates. We revised the Results, Discussion, and Table 3 footnote to explicitly state that susceptibility percentages for species with fewer than 30 isolates should be interpreted descriptively as isolate-level surveillance signals rather than as cumulative institutional susceptibility or resistance estimates.*  *We also clarified that the AFST analysis was performed at the isolate-record level. After reviewing the AFST dataset, no repeated de-identified medical-record codes were detected among the 196 records included in the susceptibility analysis. Nevertheless, because several species were represented by small denominators, the susceptibility findings are now framed as descriptive microbiological surveillance data rather than as a basis, by themselves, for defining institutional treatment protocols. The Discussion was also revised to emphasize that these data support species-level identification and isolate-level susceptibility testing in clinically significant infections but should not be used alone to define institutional treatment protocols.* | *The Results section, Discussion section, and Table 3 footnote were revised to clarify that susceptibility percentages for species with fewer than 30 isolates should be interpreted as descriptive isolate-level surveillance signals rather than representative institutional resistance rates or cumulative susceptibility estimates. The manuscript also clarifies that AFST analyses were performed at the isolate-record level and that no repeated de-identified medical-record codes were detected among the 196 AFST records. Statements suggesting direct use of these data for institutional treatment protocols were softened. Location: Results section, page 16, lines 271–280; Table 3, pages 16–18, lines 281–290; Discussion section, page 22, lines 381–391.* |
| R1-07 | Reviewer 1 | 7. Major Revision: Clinical Relevance of Pooled Susceptibility Data In Table 3, the authors pool susceptibility results from all clinical sources—including superficial (36.62%), urogenital (33.44%), and blood (7.32%)—into a single species-level analysis. This practice significantly diminishes the clinical utility of the data for a high-complexity hospital. • Confounding by Site: The selective pressure and previous antifungal exposure for mucocutaneous isolates (often outpatient) differ fundamentally from those of invasive bloodstream isolates (often ICU-acquired). • Interpretation Bias: Clinical breakpoints (CLSI/EUCAST) are site-specific. An MIC that is considered "Susceptible" for a urinary tract infection (where drugs like fluconazole concentrate) may not be appropriate for guiding the management of candidemia. • Misleading Stewardship: By allowing superficial and urinary isolates to dominate the denominator (>70% of samples), the authors may be masking critical resistance trends in the smaller bloodstream subgroup (n=23). Recommendation to Authors: To ensure the data is "practically useful" for strengthening local surveillance, the susceptibility analysis should ideally be stratified by Invasive (Sterile Site) vs. Non-Invasive (Colonization/Superficial) samples. If the sample size is too small for stratification (as established in previous comments regarding n<10), the authors must explicitly state that the pooled data cannot be used to guide empiric therapy for invasive candidiasis and should be viewed only as a general microbiological survey. | *We thank the reviewer for this important comment. We agree that pooled susceptibility data from heterogeneous clinical sources should not be interpreted as a direct guide for empiric therapy in invasive candidiasis. We revised the Results, Table 3 footnote, and Discussion to clarify that Table 3 pools isolates from different clinical sources, including superficial, urogenital, and bloodstream samples. Therefore, these data are now explicitly framed as a general microbiological survey rather than as a standalone basis for empiric treatment decisions in invasive disease.*  *We also clarified that, because the bloodstream subgroup was small and species-specific denominators were limited, stable stratification of susceptibility results by invasive versus non-invasive source would not be statistically robust. Accordingly, the pooled susceptibility findings are interpreted as descriptive isolate-level surveillance signals that support species-level identification and isolate-level susceptibility testing in clinically significant infections but should not be used alone to define institutional treatment protocols.* | *The Results section, Table 3 footnote, and Discussion section were revised to clarify that pooled susceptibility results include isolates from heterogeneous clinical sources, including superficial, urogenital, and bloodstream samples. The manuscript now states that these pooled data should be interpreted as a general microbiological survey and not as a standalone guide for empiric therapy in invasive candidiasis. The Discussion was also revised to indicate that these data should not be used alone to define institutional treatment protocols.* *Location: Results section, page 16, lines 277–280; Table 3, pages 16–18, lines 281–290; Discussion section, page 22, lines 386–390.* |
| R1-08 | Reviewer 1 | 1. Nomenclature Inconsistency: In Table 2 (Page 12) and Table 3 (Page 14), the term "no-albicans" is used. However, the correct scientific term "non-albicans" is used in the Abstract and Conclusion. Please standardize this to "non-albicans" throughout the manuscript. | *We thank the reviewer for noting this nomenclature inconsistency. We reviewed the manuscript and standardized the correct term “non-albicans” throughout the text, tables, and footnotes. The incorrect form “no-albicans” was corrected wherever it appeared.* | *The incorrect term “no-albicans” was corrected to the scientifically appropriate term “non-albicans” throughout the manuscript. The terminology was standardized in the text paragraphs, Table 2, Table 3, table footnotes, and all related descriptions of non-albicans Candida isolates.* *Location: throughout the manuscript, including Abstract, page 2, lines 37–38; Keywords, page 3, lines 40–41; Introduction, page 3, line 51; Results, pages 14–16; Discussion, pages 20–22; Conclusions, page 23, lines 404–418; and Tables 2–3.* |
| R1-09 | Reviewer 1 | 2. Typo: Page 16, Line 248—"prioritise" is used; ensure consistent American or British English spelling throughout based on journal preference (PLOS ONE typically prefers American English: "prioritize"). | *We thank the reviewer for noting this spelling inconsistency. We revised the manuscript to use consistent American English spelling throughout. Specifically, “prioritise” was corrected to “prioritize,” and the manuscript was reviewed for similar British/American spelling inconsistencies.* | *The spelling was standardized to American English throughout the manuscript. The term “prioritise” was corrected to “prioritize.”Location: Discussion section, page 19, lines 329–331; Results section, page 14, lines 241–243; Discussion section, page 22, lines 391–396.* |
| R1-10 | Reviewer 1 | Conclusion of Review: Mismatch Between Data Density and Clinical Recommendations  The manuscript provides a valuable baseline for Candida diversity in Tolima, Colombia; however, there is a significant mismatch between the low density of the invasive disease data and the broadness of the clinical conclusions. • Weak Clinical Utility: The reliance on pooled susceptibility data from disparate anatomical sites (urine, skin, and blood) makes the individual case signals rather than statistically sound institutional trends. • Underestimation of Burden: The reported findings unsuitable for guiding empiric therapy in high-complexity units. • Statistical Fragility: The "non-albicans diversity" highlighted in the results is based on species counts so small (n=1 to 9) that they represent candidemia rate (0.08 per 1,000 admissions) suggests a systemic under-capture of invasive cases, which may bias the identified risk factors. Final Recommendation: The authors must significantly temper their conclusions. References to "informing local protocols" or "guiding therapy" should be replaced with a more cautious interpretation of the data as a "longitudinal microbiological survey" with recognized limitations in invasive disease capture. | *We thank the reviewer for this important final assessment. We agree that the clinical interpretation of the findings should be tempered given the low number of recorded invasive disease cases, the use of separate non-linked clinical/administrative and microbiological data sources, the small species-specific denominators, and the pooled nature of the susceptibility dataset across heterogeneous clinical specimens. We revised the Conclusions to frame the study as a longitudinal clinical–microbiological survey and local surveillance baseline for Candida infections, rather than as a direct basis for empiric therapy or institutional treatment protocols.*  *Statements suggesting susceptibility-guided therapy, direct guidance of invasive disease management, or use of the findings to inform local treatment protocols were replaced with more cautious wording focused on species-level identification, isolate-level susceptibility testing, improved microbiological confirmation, and descriptive surveillance.* | *The Conclusions were revised to significantly temper the clinical interpretation of the findings. Statements referring to susceptibility-guided therapy, guiding invasive disease management, or informing local treatment protocols were replaced with cautious wording that frames the study as a longitudinal clinical–microbiological survey and local surveillance baseline. The revised conclusion now emphasizes limitations related to low recorded candidemia counts, separate non-linked clinical/administrative and microbiological data sources, pooled susceptibility data from heterogeneous clinical specimens, and small species-specific denominators. Location: Discussion section, page 22, lines 381–402; Conclusions section, page 23, lines 404–419.* |

4. Reviewer 2 comments – matrix to complete

| **N°** | **Source** | **Exact comment or request** | **Author's response** | **Change made / new page and line** |
| --- | --- | --- | --- | --- |
| R2-01 | Reviewer 2 | Abstract: Please clarify the study setting → mentions 'mid-sized departments' in the background, but focuses on a 'high-complexity hospital' in the methods; please ensure these terms accurately reflect the scope your research | *We thank the reviewer for this observation. We revised the Abstract to clarify the study setting and avoid ambiguity in the term “department.” In the revised version, we specify that the evidence gap refers to underrepresented Colombian administrative regions such as Tolima, while the study itself was conducted in a high-complexity hospital in Ibagué, Tolima, Colombia. This distinction clarifies that the manuscript reports hospital-based data from a specific Colombian administrative region, rather than department-wide surveillance data.* | *The Abstract was revised to clarify the study setting. The term “mid-sized Colombian departments” was replaced with “underrepresented Colombian administrative regions such as Tolima,” and the Methods sentence specifies that the study was conducted in a high-complexity hospital in Ibagué, Tolima, Colombia. Location: Abstract, page 2, lines 19–24.* |
| R2-02 | Reviewer 2 | The keywords are highly redundant (e.g., Candida, candidiasis, candidemia). Please optimize to enhance the article's discoverability | *Thank you for this suggestion. We agree that the previous keywords were redundant. The keyword list has been revised to reduce overlap and improve the article’s discoverability by using more specific terms related to the study scope, including candidemia, non-albicans Candida, antifungal susceptibility, hospital epidemiology, and Colombia.* | *Change made: The keywords were updated as follows: Candida infections; candidemia; non-albicans Candida; antifungal susceptibility; hospital epidemiology; Colombia.*  *Location: Keywords, page 3, lines 40–41.* |
| R2-03 | Reviewer 2 | Please define the acronym SIVIGILA upon its first mention in the introduction | *We thank the reviewer for this observation. We revised the Introduction to define SIVIGILA at first mention as Colombia’s National Public Health Surveillance System, while indicating that the acronym corresponds to its Spanish name. We also clarified recent actions led by the Colombian National Institute of Health, including the incorporation of C. auris into SIVIGILA, the updating of national guidelines, and laboratory surveillance criteria for Candida spp. isolates recovered from healthcare-associated infections (HAIs; IAAS in Spanish) or showing antifungal resistance profiles.* | *The acronym SIVIGILA was defined at first mention in the Introduction as Colombia’s National Public Health Surveillance System. The sentence was also revised to acknowledge recent actions led by the Colombian National Institute of Health in fungal disease surveillance, including national guideline updates and laboratory surveillance criteria for Candida spp. isolates. Location: Introduction, page 3, lines 57–60; page 4, lines 61–63..* |
| R2-04 | Reviewer 2 | Introduction → The objective of this study → Please clarify the study setting. “Department” refers to a geographical region in Colombia; to avoid confusion with clinical departments, please explicitly state whether this is a single-center study. Furthermore, instead of 'high-complexity hospital', I suggest using 'tertiary care referral center' to better reflect the facility's role in the region. | *We thank the reviewer for this clarification. We revised the objective statement in the Introduction to explicitly indicate that this was a single-center study. We also clarified that the “Colombian department of Tolima” refers to a geographical and administrative region of Colombia, not to a clinical department within the hospital. To better reflect the facility’s role while preserving the Colombian healthcare classification, we revised the wording to describe the setting as a high-complexity hospital serving as a regional referral center in Ibagué, Tolima, Colombia.* | *The objective statement in the Introduction was revised to clarify that the study was single-center and conducted in a high-complexity hospital serving as a regional referral center in Ibagué, in the Colombian department of Tolima. The wording was adjusted to avoid confusion between “department” as a Colombian geographical/administrative region and clinical departments within a hospital. Location: Introduction, page 4, lines 66–69.* |
| R2-05 | Reviewer 2 | Statistical analysis → The methodology for data management is unclear. Please revise the sentence to clearly state how data were prepared and managed before statistical analysis. For example: 'Data were managed in Microsoft Excel and analyzed using R (version 4.4.2)' | *We thank the reviewer for this suggestion. We revised the Statistical analysis section to clarify how data were prepared and managed before analysis. Specifically, we now state that data were prepared, cleaned, coded, and managed in Microsoft Excel before statistical analysis, and that all statistical analyses were performed using R software, version 4.4.2.* | *The Statistical analysis section was revised to clarify the data management workflow before analysis. The manuscript now states that data were prepared, cleaned, coded, and managed in Microsoft Excel, and analyzed using R software, version 4.4.2. Location: Statistical analysis section, page 6, lines 122–124.* |
| R2-06 | Reviewer 2 | Results → 243 013 hospitalizations were recorded → seems exceptionally high for a single-center study. Please clarify whether this number represents the total hospitalizations or what? | *We thank the reviewer for requesting this clarification. We revised the Results section to clarify that 243,013 refers to cumulative institutional care records registered in the hospital administrative database across different service locations during the full 2014–2024 study period. This value does not represent unique patients. We also clarified that this administrative denominator was used to estimate the recorded candidiasis burden.* | *The Results section was revised to clarify that 243,013 corresponds to cumulative institutional care records across different service locations during 2014–2024, rather than unique patients. The sentence was adjusted to specify that this administrative denominator was used to estimate the recorded candidiasis burden. Location: Results section, page 8, lines 150–155.* |
| R2-07 | Reviewer 2 | Figure 1A & B → The numbers displayed on the trend line are ambiguous | *We thank the reviewer for noting this ambiguity. We revised the Figure 1 legend to clarify the meaning of the numbers displayed in panels A and B. The revised legend now specifies that, in both panels, bars and their numeric labels represent annual counts, whereas the line and its numeric labels represent cumulative counts over time.* | *The Figure 1 legend was revised to clarify that, in both panels, bars and their numeric labels represent annual counts, whereas the line and its numeric labels represent cumulative counts over time. Location: Fig 1 legend, page 8, lines 160–165.* |
| R2-08 | Reviewer 2 | Results → The classification of invasive candidiasis (IC) presented in Figure 2 and the Results section is inconsistent with standard clinical definitions. IC is generally categorized into candidemia, deep-seated (non-candidemic) candidiasis, or combined/disseminated infections. Currently, the distinction between 'meningitis' and 'localized' is ambiguous and does not follow recognized clinical classifications. Please revise the categorization to reflect standard definitions to ensure clinical accuracy and comparability with existing literature. Furthermore, ensure that the definition of each category is clearly stated in the Methods/Operational Definitions section. | *We thank the reviewer for this important clarification. We agree that the previous distinction between “localized invasive” and “meningitis” could be clinically ambiguous and not fully aligned with standard invasive candidiasis classifications. We revised the Methods/Operational definitions section to clearly define the clinical categories used in the study.*  *In the revised version, clinical forms were grouped as mucocutaneous/localized non-invasive candidiasis, candidemia, deep-seated non-candidemic invasive candidiasis, or other/unspecified candidiasis. CNS involvement/meningitis was included within deep-seated non-candidemic invasive candidiasis when no candidemia diagnosis was recorded. We also clarified that combined or disseminated candidiasis was not assigned as a separate category because this condition was not documented in the clinical/administrative diagnosis fields. Figure 2 and the corresponding Results text were revised accordingly to improve clinical accuracy and comparability with existing literature.* | *The Methods/Operational definitions section was revised to define the clinical categories used for candidiasis classification, including mucocutaneous/localized non-invasive candidiasis, candidemia, deep-seated non-candidemic invasive candidiasis, and other/unspecified candidiasis. Figure 2 and the Results section were revised to align the presentation of invasive candidiasis with these definitions. The ambiguous “localized invasive” label was replaced with deep-seated non-candidemic invasive candidiasis, and CNS involvement/meningitis was incorporated within this category. Location: Methods/Operational definitions section, page 5, lines 99–105; Results section and Fig 2 legend, page 9, lines 168–177.* |
| R2-09 | Reviewer 2 | The presence of an 'Other/Unspecified' category that is nearly equal in size to the 'Localized' category is highly problematic. This indicates a substantial limitation in data granularity or diagnostic reporting | *We thank the reviewer for highlighting this important limitation. We agree that the size of the other/unspecified category reflects limited diagnostic granularity in the retrospective clinical/administrative records. We revised the Results and Discussion sections to make this limitation explicit.*  *In the revised version, we clarify that the other/unspecified group should not be interpreted as a distinct clinical syndrome, but rather as a category derived from incomplete or non-specific diagnostic information in the clinical/administrative database. We also indicate that this limitation may have contributed to misclassification or under-recognition of more specific clinical forms, particularly when the anatomical site or invasive status was not clearly documented.* | *The Results section was revised to clarify that the other/unspecified group mainly reflected unspecified candidiasis records and should be interpreted as evidence of limited diagnostic granularity in the retrospective clinical/administrative database rather than as a distinct clinical syndrome. The Discussion was also revised to explicitly acknowledge this category as an important limitation that may have contributed to misclassification or under-recognition of more specific clinical forms. Location: Results section, page 9, lines 170–174; Discussion section, page 20, lines 341–345.* |
| R2-10 | Reviewer 2 | The lack of cross-database linkage severely undermines the validity of the multivariable analysis. A multivariable model requires patient-level data to accurately establish independent risk factors (e.g., associating ICU stay with confirmed candidemia in the same individual). As the clinical and microbiological datasets were analyzed separately without individual-patient linkage, the study cannot establish causal or associative relationships between clinical risk factors and the presence of Candida. Consequently, any claims regarding 'established risk profiles' based on this multivariable analysis are statistically flawed and should be removed or completely re-evaluated | *We thank the reviewer for this important methodological concern. We agree that the lack of record-by-record linkage between the clinical/administrative and microbiological databases limits patient-level clinical–microbiological inference. We revised the manuscript to clarify that the multivariable models were exploratory and data-source specific, rather than definitive patient-level clinical–microbiological risk models.*  *Specifically, the candidemia and invasive candidiasis models were based on clinically recorded outcomes in the clinical/administrative database, whereas the non-albicans model was based on isolate-level records in the microbiological database. We removed or softened claims referring to definitive “risk factors” or “established risk profiles” and replaced them with more cautious wording such as “exploratory associations,” “data-source-specific associations,” and “associated factors.” We also clarified that these models do not establish causal relationships or patient-level clinical–microbiological predictors of laboratory-confirmed Candida infection.* | *The Statistical analysis, Results, and Discussion sections were revised to clarify that the multivariable models were exploratory and data-source specific. The manuscript now states that the candidemia and invasive candidiasis models were based on the clinical/administrative database, whereas the non-albicans model was based on microbiological isolate-level records. Claims suggesting definitive risk factors or patient-level clinical–microbiological inference were softened and replaced with cautious wording indicating exploratory associations. Location: Statistical analysis section, pages 6–7, lines 128–136; Results section, pages 14–15, lines 247–258; Discussion section, page 21, lines 365–375..* |
| R2-11 | Reviewer 2 | The dataset covering 2014–2024 is substantial and holds great potential. However, the current manuscript attempts to cover too broad a scope, resulting in superficial analyses that do not fully leverage the data's potential. To enhance the scientific rigor of this work, I recommend the following: 1. Re-focus the scope: Please select one primary research question (e.g., epidemiological shifts of Candida species over the decade or a deep-dive analysis into clinical risk factors) rather than attempting a comprehensive overview that compromises depth. 2. Refine Operational Definitions: The current definitions, especially regarding IC subtypes (e.g., 'Other/Unspecified' vs 'Localized'), are not clinically standardized. Please align these with current guidelines (e.g., IDSA/ESCMID). 3. Prioritize Data Quality over Quantity: It is better to provide a robust analysis of a subset of well-linked patient data than a wide, superficial analysis of poorly linked datasets. Consider focusing the analytical part of the study only on the period where data linkage and MIC information are reliable (e.g., 2022–2024). | *We thank the reviewer for this important overall recommendation. We agree that the original framing of the manuscript could suggest a broader clinical inference than supported by the available retrospective data. We therefore revised the manuscript to re-focus the study as a longitudinal clinical–microbiological survey of Candida infections in a single high-complexity hospital in Tolima, rather than as a definitive patient-level risk-factor analysis.*  *The revised manuscript now emphasizes recorded clinical burden, species distribution, descriptive microbiological trends, exploratory data-source-specific associations, and antifungal susceptibility patterns. We clarified that the clinical/administrative and microbiological databases were not linked record by record, that multivariable models were exploratory and data-source specific, and that MIC-based susceptibility analyses were restricted to 2022–2024, the period with structured MIC data. We also refined the operational definitions for invasive candidiasis, including candidemia and deep-seated non-candidemic invasive candidiasis, and explicitly acknowledged the limitation represented by other/unspecified records.*  *We preserved the 2014–2024 longitudinal scope because this period captures a relevant transition in institutional microbiological capacity, including the later incorporation of automated identification/susceptibility testing and structured WHONET MIC exports. Accordingly, claims suggesting definitive risk factors, causal inference, susceptibility-guided therapy, or direct use of the findings to define institutional protocols were removed or substantially tempered.* | *The manuscript was revised to re-focus the study as a longitudinal clinical–microbiological survey and descriptive local surveillance baseline rather than a definitive patient-level risk-factor or treatment-guidance study. The Introduction, Methods, Results, Discussion, and Conclusions were revised to clarify the single-center scope, the source-specific nature of the analyses, the lack of record-by-record database linkage, the restriction of MIC-based susceptibility analyses to 2022–2024, and the updated operational definitions for invasive candidiasis. Claims regarding definitive risk factors, causal inference, susceptibility-guided therapy, or use of the findings to define institutional protocols were softened and replaced with cautious wording focused on exploratory associations, species-level identification, isolate-level susceptibility testing, microbiological confirmation, and local surveillance. Location: Introduction, page 4, lines 64–69; Methods, pages 4–7, lines 73–136; Results, pages 8–18, lines 148–297; Discussion, pages 18–22, lines 299–402; Conclusions, page 23, lines 404–418.* |
