# Supplementary material for: Clinical and microbiological epidemiology of Candida infections in a high-complexity hospital in Tolima, Colombia (2014–2024)
Source: PLoS One. 2026 Jul 24;21(7):e0354684. doi: 10.1371/journal.pone.0354684 (PMC13399354; doi:10.1371/journal.pone.0354684)
Supplement: Peer review history

# Peer review: Decision Letter 0

**DOI:** 10.1371/journal.pone.0354684.r001

**Authors:** Theerapong Krajaejun

26 May 2026

PONE-D-26-11229

Clinical and microbiological epidemiology of Candida infections in a high-complexity hospital in Tolima, Colombia (2014–2024)

PLOS One

Dear Dr. Gonzalez,

Thank you for submitting your manuscript to PLOS ONE. After careful consideration, we feel that it has merit but does not fully meet PLOS ONE’s publication criteria as it currently stands. Therefore, we invite you to submit a revised version of the manuscript that addresses the points raised during the review process.

Please submit your revised manuscript by Jul 10 2026 11:59PM. If you will need more time than this to complete your revisions, please reply to this message or contact the journal office at plosone@plos.org. When you're ready to submit your revision, log on to https://www.editorialmanager.com/pone/ and select the 'Submissions Needing Revision' folder to locate your manuscript file.

Please include the following items when submitting your revised manuscript:

- A letter that responds to each point raised by the academic editor and reviewer(s). You should upload this letter as a separate file labeled 'Response to Reviewers'.
- A marked-up copy of your manuscript that highlights changes made to the original version. You should upload this as a separate file labeled 'Revised Manuscript with Track Changes'.
- An unmarked version of your revised paper without tracked changes. You should upload this as a separate file labeled 'Manuscript'.

If you would like to make changes to your financial disclosure, please include your updated statement in your cover letter. Guidelines for resubmitting your figure files are available below the reviewer comments at the end of this letter.

If applicable, we recommend that you deposit your laboratory protocols in protocols.io to enhance the reproducibility of your results. Protocols.io assigns your protocol its own identifier (DOI) so that it can be cited independently in the future. For instructions see: https://journals.plos.org/plosone/s/submission-guidelines#loc-laboratory-protocols. Additionally, PLOS ONE offers an option for publishing peer-reviewed Lab Protocol articles, which describe protocols hosted on protocols.io. Read more information on sharing protocols at https://plos.org/protocols?utm_medium=editorial-email&utm_source=authorletters&utm_campaign=protocols.

As the corresponding author, your ORCID iD is verified in the submission system and will appear in the published article. PLOS supports the use of ORCID, and we encourage all coauthors to register for an ORCID iD and use it as well. Please encourage your coauthors to verify their ORCID iD within the submission system before final acceptance, as unverified ORCID iDs will not appear in the published article. *Only*  the individual author can complete the verification step; PLOS staff *cannot*  verify ORCID iDs on behalf of authors.

We look forward to receiving your revised manuscript.

Kind regards,

Theerapong Krajaejun, M.D.

Academic Editor

PLOS One

Journal Requirements:

When submitting your revision, we need you to address these additional requirements.

1. Please ensure that your manuscript meets PLOS ONE's style requirements, including those for file naming. The PLOS ONE style templates can be found at https://journals.plos.org/plosone/s/file?id=wjVg/PLOSOne_formatting_sample_main_body.pdf and https://journals.plos.org/plosone/s/file?id=ba62/PLOSOne_formatting_sample_title_authors_affiliations.pdf

2. Please note that PLOS One has specific guidelines on code sharing for submissions in which author-generated code underpins the findings in the manuscript. In these cases, we expect all author-generated code to be made available without restrictions upon publication of the work. Please review our guidelines at https://journals.plos.org/plosone/s/materials-and-software-sharing#loc-sharing-code and ensure that your code is shared in a way that follows best practice and facilitates reproducibility and reuse.

3. We note that you have indicated that there are restrictions to data sharing for this study. For studies involving human research participant data or other sensitive data, we encourage authors to share de-identified or anonymized data. However, when data cannot be publicly shared for ethical reasons, we allow authors to make their data sets available upon request. For information on unacceptable data access restrictions, please see http://journals.plos.org/plosone/s/data-availability#loc-unacceptable-data-access-restrictions.

Before we proceed with your manuscript, please address the following prompts:

a) If there are ethical or legal restrictions on sharing a de-identified data set, please explain them in detail (e.g., data contain potentially identifying or sensitive patient information, data are owned by a third-party organization, etc.) and who has imposed them (e.g., a Research Ethics Committee or Institutional Review Board, etc.). Please also provide contact information for a data access committee, ethics committee, or other institutional body to which data requests may be sent.

b) If there are no restrictions, please upload the minimal anonymized data set necessary to replicate your study findings to a stable, public repository and provide us with the relevant URLs, DOIs, or accession numbers. Please see http://www.bmj.com/content/340/bmj.c181.long for guidelines on how to de-identify and prepare clinical data for publication. For a list of recommended repositories, please see https://journals.plos.org/plosone/s/recommended-repositories. You also have the option of uploading the data as Supporting Information files, but we would recommend depositing data directly to a data repository if possible.

Please update your Data Availability statement in the submission form accordingly.

3. Your ethics statement should only appear in the Methods section of your manuscript. If your ethics statement is written in any section besides the Methods, please move it to the Methods section and delete it from any other section. Please ensure that your ethics statement is included in your manuscript, as the ethics statement entered into the online submission form will not be published alongside your manuscript.

If the reviewer comments include a recommendation to cite specific previously published works, please review and evaluate these publications to determine whether they are relevant and should be cited. There is no requirement to cite these works unless the editor has indicated otherwise.

Additional Editor Comments:

The reviewers have evaluated this 10-year study on Candida infections in Tolima, Colombia. Key concerns include the need for clearer correlations between clinical and microbiological data, an explicit description of antifungal susceptibility testing methods, and clarification of discrepancies in candidemia incidence rates relative to national averages. Additionally, the study’s broad scope appears to limit the depth of analysis in specific areas. Overall, while the study provides valuable insights, it would benefit from greater methodological clarity and improved data analysis.

[Note: HTML markup is below. Please do not edit.]

Reviewers' comments:

Reviewer's Responses to Questions

**Comments to the Author**

1. Is the manuscript technically sound, and do the data support the conclusions?

The manuscript must describe a technically sound piece of scientific research with data that supports the conclusions. Experiments must have been conducted rigorously, with appropriate controls, replication, and sample sizes. The conclusions must be drawn appropriately based on the data presented.

Reviewer #1: Partly

Reviewer #2: Partly

**********

2. Has the statistical analysis been performed appropriately and rigorously?

Reviewer #1: Yes

Reviewer #2: No

**********

3. Have the authors made all data underlying the findings in their manuscript fully available?

The PLOS Data policy requires authors to make all data underlying the findings described in their manuscript fully available without restriction, with rare exception (please refer to the Data Availability Statement in the manuscript PDF file). The data should be provided as part of the manuscript or its supporting information, or deposited to a public repository. For example, in addition to summary statistics, the data points behind means, medians and variance measures should be available. If there are restrictions on publicly sharing data—e.g. participant privacy or use of data from a third party—those must be specified.

Reviewer #1: Yes

Reviewer #2: Yes

**********

4. Is the manuscript presented in an intelligible fashion and written in standard English?

PLOS ONE does not copyedit accepted manuscripts, so the language in submitted articles must be clear, correct, and unambiguous. Any typographical or grammatical errors should be corrected at revision, so please note any specific errors here.

Reviewer #1: Yes

Reviewer #2: Yes

**********

5. Review Comments to the Author

Please use the space provided to explain your answers to the questions above. You may also include additional comments for the author, including concerns about dual publication, research ethics, or publication ethics. (Please upload your review as an attachment if it exceeds 20,000 characters)

Reviewer #1: Peer Review Report:

General Assessment

This 10-year longitudinal study provides essential epidemiological data on Candida infections in Tolima, Colombia. The authors successfully integrated clinical and microbiological databases to characterize a sustained institutional burden. The statistical approach is robust, employing Firth-penalized regression to address the low frequency of candidemia events. The commitment to open science, demonstrated by the inclusion of Zenodo and GitHub repositories for code and data, is commendable.

Major Comments for Improvement

1. Database Linkage and Clinical Correlation

The authors explicitly state that the clinical and microbiological databases were analyzed separately without individual-patient level linkage. While this is a common limitation in retrospective studies, it creates a gap in correlating clinical syndromes directly with specific fungal etiologies. Interestingly, the clinical database identified 20 candidemia cases , while the microbiological database identified 23 blood isolates.

Recommendation: To improve the technical soundness of the "Associated Factors" section (Page 11, Lines 177–184), the authors should clarify if the 20 candidemia cases used for the multivariable model are subset from the same patient group as the 23 blood isolates. If linkage was performed for this specific subset, it should be stated.

2. 5-Fluorocytosine (5-FC) Susceptibility Anomaly

Table 3 (Page 13) shows that 99.06% (106/107) of C. albicans isolates were classified as Non-Wild Type (NWT) for 5-FC. As the authors correctly note in the Discussion (Page 18, Lines 291–296), C. albicans is typically highly susceptible to 5-FC. Attributing this to a "surveillance artifact" related to system configuration or cutoff availability is a fair assessment.

Recommendation: To ensure this doesn't mislead clinical readers, the authors should specify the VITEK 2 AST card version used and confirm if WHONET expert rules were applied. If the data is an artifact, consider moving the 5-FC results to a Supplementary Table to keep the focus of Table 3 on clinically relevant susceptibilities.

3. Temporal Trend Interpretation

Figure 1A (Page 28) and line 113 (Page 6) identify a peak in 2018 followed by a significant decline through 2024. In the Discussion (Page 16, Lines 241–242), the authors suggest this might reflect changes in coding or clinical criteria rather than true incidence.

Recommendation: Given the administrative nature of the clinical database, the authors should explicitly state if there were changes to the Hospital Information System (HIS) or a shift from ICD-9 to ICD-10 coding during the 2018–2019 period that might have impacted case capture.

4. Statistical Transparency in Regression Models

For Table 2 (Page 12), please include the final N (number of cases included) in the footer for each model (Candidemia, Invasive Candidiasis, and Non-albicans isolation). This allows reviewers to verify the ratio of events to independent variables.

5. Major Concern: Candidemia Incidence and Database Discrepancy

The study reports 20 candidemia episodes identified in the clinical database and 23 blood isolates identified in the microbiological database over an 11-year period (2014–2024) across 243,013 hospitalizations. This results in an incidence rate of approximately 0.08 per 1,000 admissions, which is significantly lower than national Colombian averages (~1.96 per 1,000) and regional Latin American benchmarks (0.74 to 6.0 per 1,000 admissions). I have the following concerns and requests for clarification:

• Internal Consistency: Why is there a discrepancy between the 20 clinical episodes and the 23 laboratory isolates? Does this imply that three confirmed laboratory cases were not captured in the administrative/clinical records? Please clarify if any manual validation was performed to ensure that the 20 cases used in the multivariable model represent the full clinical reality of the hospital.

• Definition of "High-Complexity": The extremely low incidence (less than 2 cases per year) seems atypical for a high-complexity facility. To provide better context for these numbers, please include specific institutional data in the Methods section, such as the total number of ICU beds and the presence of specialized services (e.g., oncology, transplantation, or neonatal ICU) during the study period.

• Information Bias: While the authors mention that fungal infections are underreported due to a lack of mandatory notification , the low count suggests a potential "information bias" stemming from the reliance on administrative ICD-10 coding. Please expand the Study Limitations to discuss how this specific reliance might have led to an underestimation of the invasive disease burden and how it may impact the power of the multivariable risk factor analysis.

6. Major Concern: Statistical Validity of Antifungal Susceptibility Testing (AFST)

While the authors provide MIC data for 196 isolates, the sample sizes for several high-priority species are critically low, which introduces a high risk of selection bias and limits the validity of the reported resistance percentages.

• Species-Level Fragility: In Table 3, susceptibility profiles for species like Nakaseomyces glabratus (n=19), Candida dubliniensis (n=4), and Wickerhamomyces anomalus (n=3) are based on numbers too small to represent institutional or regional trends. E.g. For W. anomalus, a single resistant isolate results in a reported resistance rate of 33.33%.

• Bloodstream Etiology: The bloodstream subgroup (n=23) is even more fragmented, with only one C. auris isolate and two N. glabratus isolates identified.

• Impact on Guidelines: The manuscript concludes that these findings support "susceptibility-guided therapy" and inform "local protocols". However, basing institutional protocols on such a limited number of isolates is statistically risky.

Recommendation to Authors: Please include a clear disclaimer in the Results and Discussion stating that the susceptibility percentages for species with n < 30 (consistent with CLSI M101 guidelines for cumulative antibiograms) should be interpreted as individual case signals rather than representative institutional resistance rates. The authors should also clarify if these isolates were collected from the same patient (duplicate isolates) or represent unique infection episodes.

7. Major Revision: Clinical Relevance of Pooled Susceptibility Data

In Table 3, the authors pool susceptibility results from all clinical sources—including superficial (36.62%), urogenital (33.44%), and blood (7.32%)—into a single species-level analysis. This practice significantly diminishes the clinical utility of the data for a high-complexity hospital.

• Confounding by Site: The selective pressure and previous antifungal exposure for mucocutaneous isolates (often outpatient) differ fundamentally from those of invasive bloodstream isolates (often ICU-acquired).

• Interpretation Bias: Clinical breakpoints (CLSI/EUCAST) are site-specific. An MIC that is considered "Susceptible" for a urinary tract infection (where drugs like fluconazole concentrate) may not be appropriate for guiding the management of candidemia.

• Misleading Stewardship: By allowing superficial and urinary isolates to dominate the denominator (>70% of samples), the authors may be masking critical resistance trends in the smaller bloodstream subgroup (n=23).

Recommendation to Authors: To ensure the data is "practically useful" for strengthening local surveillance, the susceptibility analysis should ideally be stratified by Invasive (Sterile Site) vs. Non-Invasive (Colonization/Superficial) samples. If the sample size is too small for stratification (as established in previous comments regarding n<10), the authors must explicitly state that the pooled data cannot be used to guide empiric therapy for invasive candidiasis and should be viewed only as a general microbiological survey.

Minor Comments

1. Nomenclature Inconsistency: In Table 2 (Page 12) and Table 3 (Page 14), the term "no-albicans" is used. However, the correct scientific term "non-albicans" is used in the Abstract and Conclusion. Please standardize this to "non-albicans" throughout the manuscript.

2. Typo: Page 16, Line 248—"prioritise" is used; ensure consistent American or British English spelling throughout based on journal preference (PLOS ONE typically prefers American English: "prioritize").

Conclusion of Review: Mismatch Between Data Density and Clinical Recommendations

The manuscript provides a valuable baseline for Candida diversity in Tolima, Colombia; however, there is a significant mismatch between the low density of the invasive disease data and the broadness of the clinical conclusions.

• Weak Clinical Utility: The reliance on pooled susceptibility data from disparate anatomical sites (urine, skin, and blood) makes the individual case signals rather than statistically sound institutional trends.

• Underestimation of Burden: The reported findings unsuitable for guiding empiric therapy in high-complexity units.

• Statistical Fragility: The "non-albicans diversity" highlighted in the results is based on species counts so small (n=1 to 9) that they represent candidemia rate (0.08 per 1,000 admissions) suggests a systemic under-capture of invasive cases, which may bias the identified risk factors.

Final Recommendation: The authors must significantly temper their conclusions. References to "informing local protocols" or "guiding therapy" should be replaced with a more cautious interpretation of the data as a "longitudinal microbiological survey" with recognized limitations in invasive disease capture.

Reviewer #2: Abstract: Please clarify the study setting mentions 'mid-sized departments' in the background, but focuses on a 'high-complexity hospital' in the methods; please ensure these terms accurately reflect the scope your research

The keywords are highly redundant (e.g., Candida, candidiasis, candidemia). Please optimize to enhance the article's discoverability

Please define the acronym SIVIGILA upon its first mention in the introduction

Introduction The objective of this study Please clarify the study setting. “Department” refers to a geographical region in Colombia; to avoid confusion with clinical departments, please explicitly state whether this is a single-center study. Furthermore, instead of 'high-complexity hospital', I suggest using 'tertiary care referral center' to better reflect the facility's role in the region.

Statistical analysis The methodology for data management is unclear. Please revise the sentence to clearly state how data were prepared and managed before statistical analysis. For example: 'Data were managed in Microsoft Excel and analyzed using R (version 4.4.2)

Results 243 013 hospitalizations were recorded seems exceptionally high for a single-center study. Please clarify whether this number represents the total hospitalizations or what?

Figure 1A & B The numbers displayed on the trend line are ambiguous

Results The classification of invasive candidiasis (IC) presented in Figure 2 and the Results section is inconsistent with standard clinical definitions. IC is generally categorized into candidemia, deep-seated (non-candidemic) candidiasis, or combined/disseminated infections.Currently, the distinction between 'meningitis' and 'localized' is ambiguous and does not follow recognized clinical classifications. Please revise the categorization to reflect standard definitions to ensure clinical accuracy and comparability with existing literature. Furthermore, ensure that the definition of each category is clearly stated in the Methods/Operational Definitions section.

The presence of an 'Other/Unspecified' category that is nearly equal in size to the 'Localized' category is highly problematic. This indicates a substantial limitation in data granularity or diagnostic reporting

The lack of cross-database linkage severely undermines the validity of the multivariable analysis. A multivariable model requires patient-level data to accurately establish independent risk factors (e.g., associating ICU stay with confirmed candidemia in the same individual).

As the clinical and microbiological datasets were analyzed separately without individual-patient linkage, the study cannot establish causal or associative relationships between clinical risk factors and the presence of Candida. Consequently, any claims regarding 'established risk profiles' based on this multivariable analysis are statistically flawed and should be removed or completely re-evaluated

The dataset covering 2014–2024 is substantial and holds great potential. However, the current manuscript attempts to cover too broad a scope, resulting in superficial analyses that do not fully leverage the data's potential.To enhance the scientific rigor of this work, I recommend the following:

1. Re-focus the scope: Please select one primary research question (e.g., epidemiological shifts of Candida species over the decade or a deep-dive analysis into clinical risk factors) rather than attempting a comprehensive overview that compromises depth.

2. Refine Operational Definitions: The current definitions, especially regarding IC subtypes (e.g., 'Other/Unspecified' vs 'Localized'), are not clinically standardized. Please align these with current guidelines (e.g., IDSA/ESCMID).

3. Prioritize Data Quality over Quantity: It is better to provide a robust analysis of a subset of well-linked patient data than a wide, superficial analysis of poorly linked datasets. Consider focusing the analytical part of the study only on the period where data linkage and MIC information are reliable (e.g., 2022–2024)."

**********

6. PLOS authors have the option to publish the peer review history of their article (what does this mean?). If published, this will include your full peer review and any attached files.

If you choose “no”, your identity will remain anonymous but your review may still be made public.

**Do you want your identity to be public for this peer review?**  For information about this choice, including consent withdrawal, please see our Privacy Policy.

Reviewer #1: No

Reviewer #2: **Yes:**  Meiliyana Wijaya

**********

[NOTE: If reviewer comments were submitted as an attachment file, they will be attached to this email and accessible via the submission site. Please log into your account, locate the manuscript record, and check for the action link "View Attachments". If this link does not appear, there are no attachment files.]

To ensure your figures meet our technical requirements, please review our figure guidelines: https://journals.plos.org/plosone/s/figures

You may also use PLOS’s free figure tool, NAAS, to help you prepare publication quality figures: https://journals.plos.org/plosone/s/figures#loc-tools-for-figure-preparation.

NAAS will assess whether your figures meet our technical requirements by comparing each figure against our figure specifications.

# Author comment: Author response to Decision Letter 1

**DOI:** 10.1371/journal.pone.0354684.r002

26 Jun 2026

Dear Academic Editor and Reviewers,

We would like to sincerely thank you for the careful evaluation of our manuscript entitled “Clinical and microbiological epidemiology of Candida infections in a high-complexity hospital in Tolima, Colombia (2014–2024)” (PONE-D-26-11229). We are grateful for the constructive comments and recommendations, which helped us improve the clarity, methodological rigor, clinical interpretation, and overall presentation of the study. We have carefully revised the manuscript in response to each comment raised by the Academic Editor and both reviewers. In particular, we clarified the relationship between the clinical/administrative and microbiological data sources, explicitly stating that they originated from the same participating hospital but were not linked record by record. We also revised the interpretation of the multivariable models, now framing them as exploratory and data-source specific rather than as definitive patient-level clinical–microbiological risk models.

The Methods section was substantially revised to improve transparency regarding study design, operational definitions, data management, antifungal susceptibility testing, VITEK 2 card use, WHONET MIC exports, and the interpretation of CLSI/EUCAST-based susceptibility categories. We also refined the classification of invasive candidiasis to distinguish candidemia and deep-seated non-candidemic invasive candidiasis, while explicitly acknowledging the limitations associated with other/unspecified clinical records. The Results, Discussion, and Conclusions were also revised to provide a more cautious and balanced interpretation of the findings. We tempered claims regarding clinical risk factors, empiric therapy, and institutional treatment protocols, and reframed the study as a longitudinal clinical–microbiological survey and local surveillance baseline.

In addition, we updated the tables, figures, figure legends, supplementary materials, Data Availability statement, repository information, licenses, and author metadata. The revised submission package includes a point-by-point Response to Reviewers, a marked version of the revised manuscript showing the changes made, and a clean version of the manuscript. De-identified datasets, R scripts, supporting workbooks, and reproducibility materials have been made available through Zenodo and GitHub. The authors’ ORCID identifiers have also been updated as follows:

Rafael Augusto Ramírez-Trujillo: 0000-0001-9355-1765

Paula Carvajal Hernández: 0000-0002-5024-1019

Ángel González: 0000-0002-7052-7938

We believe that the revised manuscript is clearer, more methodologically transparent, and more appropriately framed in relation to the available data. We again thank the Academic Editor and reviewers for their valuable feedback and hope that the revised version adequately addresses all concerns raised during the review process.

Sincerely,

On behalf of all authors

NOTE: ALL TEH POINT-BY-POINT RESPONSES TO THE REVIEWERS ARE FOUND IN THE RESPONSE LETTER (REBUTTAL LETTER). THERE ARE NOT DESCRIBED HERE BECAUSE THEY ARE IN A COLUMN FORMAT FOR EASIER COMPARISON.

Attachment Submitted filename: Rebuttal Letter.docx

# Peer review: Decision Letter 1

**DOI:** 10.1371/journal.pone.0354684.r003

**Authors:** Theerapong Krajaejun

6 Jul 2026

PONE-D-26-11229R1

Clinical and microbiological epidemiology of Candida infections in a high-complexity hospital in Tolima, Colombia (2014–2024)

PLOS One

Dear Dr. Gonzalez,

Thank you for submitting your manuscript to PLOS ONE. After careful consideration, we feel that it has merit but does not fully meet PLOS ONE’s publication criteria as it currently stands. Therefore, we invite you to submit a revised version of the manuscript that addresses the points raised during the review process.

Please submit your revised manuscript by Aug 20 2026 11:59PM. If you will need more time than this to complete your revisions, please reply to this message or contact the journal office at plosone@plos.org. When you're ready to submit your revision, log on to https://www.editorialmanager.com/pone/ and select the 'Submissions Needing Revision' folder to locate your manuscript file.

Please include the following items when submitting your revised manuscript:

- A letter that responds to each point raised by the academic editor and reviewer(s). You should upload this letter as a separate file labeled 'Response to Reviewers'.
- A marked-up copy of your manuscript that highlights changes made to the original version. You should upload this as a separate file labeled 'Revised Manuscript with Track Changes'.
- An unmarked version of your revised paper without tracked changes. You should upload this as a separate file labeled 'Manuscript'.

If you would like to make changes to your financial disclosure, please include your updated statement in your cover letter. Guidelines for resubmitting your figure files are available below the reviewer comments at the end of this letter.

If applicable, we recommend that you deposit your laboratory protocols in protocols.io to enhance the reproducibility of your results. Protocols.io assigns your protocol its own identifier (DOI) so that it can be cited independently in the future. For instructions see: https://journals.plos.org/plosone/s/submission-guidelines#loc-laboratory-protocols. Additionally, PLOS ONE offers an option for publishing peer-reviewed Lab Protocol articles, which describe protocols hosted on protocols.io. Read more information on sharing protocols at https://plos.org/protocols?utm_medium=editorial-email&utm_source=authorletters&utm_campaign=protocols.

As the corresponding author, your ORCID iD is verified in the submission system and will appear in the published article. PLOS supports the use of ORCID, and we encourage all coauthors to register for an ORCID iD and use it as well. Please encourage your coauthors to verify their ORCID iD within the submission system before final acceptance, as unverified ORCID iDs will not appear in the published article. *Only*  the individual author can complete the verification step; PLOS staff *cannot*  verify ORCID iDs on behalf of authors.

We look forward to receiving your revised manuscript.

Kind regards,

Theerapong Krajaejun, M.D.

Academic Editor

PLOS One

Journal Requirements:

If the reviewer comments include a recommendation to cite specific previously published works, please review and evaluate these publications to determine whether they are relevant and should be cited. There is no requirement to cite these works unless the editor has indicated otherwise.

Please review your reference list to ensure that it is complete and correct. If you have cited papers that have been retracted, please include the rationale for doing so in the manuscript text, or remove these references and replace them with relevant current references. Any changes to the reference list should be mentioned in the rebuttal letter that accompanies your revised manuscript. If you need to cite a retracted article, indicate the article’s retracted status in the References list and also include a citation and full reference for the retraction notice.

**Additional Editor Comments:**

The reviewer has requested several minor revisions to improve the clarity and interpretation of the manuscript. Please carefully address the reviewer’s comments regarding terminology, variable definitions, risk factors, and the discussion of species distribution and antifungal susceptibility.

[Note: HTML markup is below. Please do not edit.]

Reviewers' comments:

Reviewer's Responses to Questions

**Comments to the Author**

1. If the authors have adequately addressed your comments raised in a previous round of review and you feel that this manuscript is now acceptable for publication, you may indicate that here to bypass the “Comments to the Author” section, enter your conflict of interest statement in the “Confidential to Editor” section, and submit your "Accept" recommendation.

Reviewer #1: All comments have been addressed

Reviewer #2: (No Response)

**********

2. Is the manuscript technically sound, and do the data support the conclusions?

The manuscript must describe a technically sound piece of scientific research with data that supports the conclusions. Experiments must have been conducted rigorously, with appropriate controls, replication, and sample sizes. The conclusions must be drawn appropriately based on the data presented.

Reviewer #1: Yes

Reviewer #2: Yes

**********

3. Has the statistical analysis been performed appropriately and rigorously?

Reviewer #1: Yes

Reviewer #2: Yes

**********

4. Have the authors made all data underlying the findings in their manuscript fully available?

The PLOS Data policy requires authors to make all data underlying the findings described in their manuscript fully available without restriction, with rare exception (please refer to the Data Availability Statement in the manuscript PDF file). The data should be provided as part of the manuscript or its supporting information, or deposited to a public repository. For example, in addition to summary statistics, the data points behind means, medians and variance measures should be available. If there are restrictions on publicly sharing data—e.g. participant privacy or use of data from a third party—those must be specified.

Reviewer #1: Yes

Reviewer #2: Yes

**********

5. Is the manuscript presented in an intelligible fashion and written in standard English?

PLOS ONE does not copyedit accepted manuscripts, so the language in submitted articles must be clear, correct, and unambiguous. Any typographical or grammatical errors should be corrected at revision, so please note any specific errors here.

Reviewer #1: Yes

Reviewer #2: Yes

**********

6. Review Comments to the Author

Please use the space provided to explain your answers to the questions above. You may also include additional comments for the author, including concerns about dual publication, research ethics, or publication ethics. (Please upload your review as an attachment if it exceeds 20,000 characters)

Reviewer #1: The authors have fully overhauled the interpretive framework of their paper. The revised manuscript is incredibly honest about its administrative and numeric boundaries, making it a highly accurate and useful baseline epidemiological survey for the Tolima region. No further revisions are required.

Reviewer #2: The previous comments have been adequately addressed by the authors. However, I would like to provide several additional comments for further improvement:

1. Abbreviations such as CLSI and EUCAST should be written in full at first mention in the abstract, as Clinical and Laboratory Standards Institute (CLSI) and European Committee on Antimicrobial Susceptibility Testing (EUCAST).

2. The formatting of fungal species names should be checked carefully and made consistent throughout the manuscript. In terms such as non-albicans Candida, the prefix “non-” should not be italicized, whereas albicans Candida should be italicized.

3. The variable socioeconomic strata appears in the results table, but its definition and interpretation are not clearly explained. The authors should clarify how this variable was categorized or scored, and whether a higher score indicates better or poorer socioeconomic status. If this variable is not further analyzed or discussed, it may be better to exclude it.

4. The section on predisposing factors should be removed or substantially revised, as these factors are not clearly included in the operational definitions and are not well explained in the discussion. For example, the term “predisposing medication” is too vague and should specify the type of medication. The discussion of short hospital stay is also unclear, particularly since most cases appear to involve mucocutaneous candidiasis, especially vulvovaginal candidiasis.

5. The discussion of Candida species distribution should be expanded. The authors should further explore the predominance of Candida albicans or non-albicans Candida species and compare these findings with previous studies.

6. The discussion of antifungal susceptibility requires clearer framing. Although many isolates appear to be susceptible to echinocandins, the discussion should not focus too heavily on invasive candidiasis if most isolates were not obtained from invasive cases. The authors should clearly distinguish the interpretation of antifungal susceptibility findings between invasive candidiasis and mucocutaneous candidiasis to avoid overstating the clinical implications.

**********

7. PLOS authors have the option to publish the peer review history of their article (what does this mean?). If published, this will include your full peer review and any attached files.

If you choose “no”, your identity will remain anonymous but your review may still be made public.

**Do you want your identity to be public for this peer review?**  For information about this choice, including consent withdrawal, please see our Privacy Policy.

Reviewer #1: No

Reviewer #2: No

**********

[NOTE: If reviewer comments were submitted as an attachment file, they will be attached to this email and accessible via the submission site. Please log into your account, locate the manuscript record, and check for the action link "View Attachments". If this link does not appear, there are no attachment files.]

To ensure your figures meet our technical requirements, please review our figure guidelines: https://journals.plos.org/plosone/s/figures

You may also use PLOS’s free figure tool, NAAS, to help you prepare publication quality figures: https://journals.plos.org/plosone/s/figures#loc-tools-for-figure-preparation.

NAAS will assess whether your figures meet our technical requirements by comparing each figure against our figure specifications.

# Author comment: Author response to Decision Letter 2

**DOI:** 10.1371/journal.pone.0354684.r004

9 Jul 2026

Dear editors and reviewers,

All the comments and suggestions were responded Point-by-point; you can find these responses in the rebuttal letter. This information was not responded here because they are in a column format.

All the best,

Attachment Submitted filename: Rebuttal Letter_R2.docx

# Editor report: Decision Letter 2

**DOI:** 10.1371/journal.pone.0354684.r005

**Authors:** Theerapong Krajaejun

13 Jul 2026

Clinical and microbiological epidemiology of Candida infections in a high-complexity hospital in Tolima, Colombia (2014–2024)

PONE-D-26-11229R2

Dear Dr. Gonzalez,

We’re pleased to inform you that your manuscript has been judged scientifically suitable for publication and will be formally accepted for publication once it meets all outstanding technical requirements.

Within one week, you’ll receive an e-mail detailing the required amendments. When these have been addressed, you’ll receive a formal acceptance letter and your manuscript will be scheduled for publication.

An invoice will be generated when your article is formally accepted. Please note, if your institution has a publishing partnership with PLOS and your article meets the relevant criteria, all or part of your publication costs will be covered. Please make sure your user information is up-to-date by logging into Editorial Manager at Editorial Manager® and clicking the ‘Update My Information' link at the top of the page. For questions related to billing, please contact billing support.

If your institution or institutions have a press office, please notify them about your upcoming paper to help maximize its impact. If they’ll be preparing press materials, please inform our press team as soon as possible -- no later than 48 hours after receiving the formal acceptance. Your manuscript will remain under strict press embargo until 2 pm Eastern Time on the date of publication. For more information, please contact onepress@plos.org.

Kind regards,

Theerapong Krajaejun, M.D.

Academic Editor

PLOS One

Additional Editor Comments (optional):

The authors have adequately addressed the reviewers’ comments, and the manuscript has been substantially improved. The study provides valuable epidemiological insights into Candida infections in Tolima, Colombia, and represents a meaningful contribution to the regional literature on candidiasis.

Reviewers' comments:

# Editor report: Acceptance letter

**DOI:** 10.1371/journal.pone.0354684.r006

**Authors:** Theerapong Krajaejun

PONE-D-26-11229R2

PLOS One

Dear Dr. Gonzalez,

I'm pleased to inform you that your manuscript has been deemed suitable for publication in PLOS One. Congratulations! Your manuscript is now being handed over to our production team.

At this stage, our production department will prepare your paper for publication. This includes ensuring the following:

* All references, tables, and figures are properly cited

* All relevant supporting information is included in the manuscript submission,

* There are no issues that prevent the paper from being properly typeset

You will receive further instructions from the production team, including instructions on how to review your proof when it is ready. Please keep in mind that we are working through a large volume of accepted articles, so please give us a few days to review your paper and let you know the next and final steps.

Lastly, if your institution or institutions have a press office, please let them know about your upcoming paper now to help maximize its impact. If they'll be preparing press materials, please inform our press team within the next 48 hours. Your manuscript will remain under strict press embargo until 2 pm Eastern Time on the date of publication. For more information, please contact onepress@plos.org.

You will receive an invoice from PLOS for your publication fee after your manuscript has reached the completed accept phase. If you receive an email requesting payment before acceptance or for any other service, this may be a phishing scheme. Learn how to identify phishing emails and protect your accounts at https://explore.plos.org/phishing.

If we can help with anything else, please email us at customercare@plos.org.

Thank you for submitting your work to PLOS ONE and supporting open access.

Kind regards,

PLOS ONE Editorial Office Staff

on behalf of

Dr. Theerapong Krajaejun

Academic Editor

PLOS One
